# Structure‐aware deep learning model for peptide toxicity prediction

**DOI:** 10.1002/pro.5076

**Published:** 2024-06-22

**Authors:** Hossein Ebrahimikondori, Darcy Sutherland, Anat Yanai, Amelia Richter, Ali Salehi, Chenkai Li, Lauren Coombe, Monica Kotkoff, René L. Warren, Inanc Birol

**Affiliations:** ^1^ Canada's Michael Smith Genome Sciences Centre BC Cancer Agency Vancouver British Columbia Canada; ^2^ Bioinformatics Graduate Program University of British Columbia Vancouver British Columbia Canada; ^3^ Public Health Laboratory British Columbia Centre for Disease Control Vancouver British Columbia Canada; ^4^ Department of Pathology and Laboratory Medicine University of British Columbia Vancouver British Columbia Canada; ^5^ Department of Medical Genetics University of British Columbia Vancouver British Columbia Canada

**Keywords:** 3D structures, antimicrobial peptides, attention mechanism, graph neural networks, multi‐modal deep learning, tAMPer

## Abstract

Antimicrobial resistance is a critical public health concern, necessitating the exploration of alternative treatments. While antimicrobial peptides (AMPs) show promise, assessing their toxicity using traditional wet lab methods is both time‐consuming and costly. We introduce tAMPer, a novel multi‐modal deep learning model designed to predict peptide toxicity by integrating the underlying amino acid sequence composition and the three‐dimensional structure of peptides. tAMPer adopts a graph‐based representation for peptides, encoding ColabFold‐predicted structures, where nodes represent amino acids and edges represent spatial interactions. Structural features are extracted using graph neural networks, and recurrent neural networks capture sequential dependencies. tAMPer's performance was assessed on a publicly available protein toxicity benchmark and an AMP hemolysis data we generated. On the latter, tAMPer achieves an F1‐score of 68.7%, outperforming the second‐best method by 23.4%. On the protein benchmark, tAMPer exhibited an improvement of over 3.0% in the F1‐score compared to current state‐of‐the‐art methods. We anticipate tAMPer to accelerate AMP discovery and development by reducing the reliance on laborious toxicity screening experiments.

## 1 INTRODUCTION

Antimicrobial resistance (AMR) poses an urgent global public health concern that requires immediate attention (Dadgostar, 2019). According to a report from the Centers for Disease Control and Prevention, about 1.27 million deaths worldwide were associated with AMR in 2019 (Murray et al., 2022). If unchecked, it is projected that the global burden of AMR could result in up to 10 million deaths annually by 2050 (O'Neill, 2014). AMR arises from random genetic changes and mutations in bacterial genomes in response to selective pressures, enabling resistance to antimicrobial agents (Gillings et al., 2017). The overuse and misuse of antibiotics over the past few decades have significantly accelerated this process by eliminating nonresistant competing bacteria (Llor & Bjerrum, 2014). To effectively combat AMR, it is essential to develop novel classes of antimicrobials that can overcome resistance mechanisms used by bacteria (Nathan, 2020).

One promising alternative to combat AMR is the use of antimicrobial peptides (AMPs) (Lewies et al., 2019), typically composed of 5–50 amino acids that naturally occur in various organisms, including humans (Wang, 2014), amphibians (Helbing et al., 2019), and plants (Broekaert et al., 1997). AMPs play a crucial role in the innate immune response of these organisms, serving as a defense mechanism against microbial infections (Wang et al., 2019). Furthermore, they exhibit broad‐spectrum antimicrobial activity, effectively combatting a wide range of pathogens, including bacteria (Brandenburg et al., 2016), fungi (De Lucca & Walsh, 1999), and even some viruses (Zhang & Gallo, 2016). This broad activity of AMPs stems from various mechanisms of action, such as direct membrane disruption and host immune response modulation (Andersson et al., 2016). These diverse mechanisms of action help to slow the development of AMR and make AMPs attractive candidates as alternatives to conventional antibiotics (Hancock & Sahl, 2006).

The interaction of AMPs with membranes is an essential component of their antimicrobial mechanism(s) of action (Nguyen et al., 2011). These interactions cannot be attributed to a specific sequential amino acid pattern or motif; instead, they originate from a combination of physicochemical and structural features (Fjell et al., 2012). Characteristics like charge, hydrophobicity, amphiphilicity, and secondary structure, are hypothesized to contribute to their antimicrobial function, and are intricately influenced by the three‐dimensional (3D) folding pattern of the peptides (Chen & Jiang, 2023; Mookherjee et al., 2020; Richter et al., 2022). AMPs are structurally diverse and can be classified into several categories based on their secondary structure, including α‐helical, β‐sheet containing, mixed, or linear extended structures (Koehbach & Craik, 2019). AMPs adopting an α‐helical structure have been reported as the most effective in interacting with bacterial membranes (Koehbach & Craik, 2019).

Conventional methods for determining peptide structures, such as nuclear magnetic resonance (NMR), X‐ray crystallography, and cryo‐electron microscopy, have historically posed challenges due to their labor‐ and time‐intensive processes, and high costs (Akdel et al., 2022). In recent years, in silico techniques for structure prediction have emerged as robust alternatives, including AlphaFold2 (AF2) (Jumper et al., 2021), which utilizes multiple sequence alignments and structural templates to predict the structure of a given peptide sequence, and ESMfold (Lin et al., 2023), which uses sequence embeddings obtained from a Protein Language Model (PLM) as inputs. These computational methods have demonstrated remarkable accuracy in predicting 3D structures that closely match experimental results (Akdel et al., 2022). Several studies have highlighted the potential of leveraging these predicted structures in diverse applications, such as protein engineering (Hsu et al., 2022) and protein function prediction (Ma et al., 2022).

In recent years, there has been a growing focus on scaling up in silico AMP discovery pipelines using computational methods for the prediction and de novo design of AMPs (Li et al., 2022; Lin et al., 2022). These computational approaches generate a large pool of AMP candidates, which must be evaluated further. One crucial aspect of this evaluation is assessing their toxicity towards host cells (Hancock & Sahl, 2006). Although there is limited understanding of the underlying mechanisms of peptide toxicity (Fjell et al., 2012), several studies have suggested exploring membrane interactions as an explanatory factor, underscoring the importance of understanding peptide structures in this context (DeGrado et al., 1982; Hollmann et al., 2016). Traditionally, the first step in evaluating peptide toxicity is to measure their hemolytic (toxicity against red blood cells) (Horváti et al., 2017) or broader cytotoxic activity (O'Brien et al., 2000). Computationally identifying potentially toxic AMP candidates prior to conducting such wet‐lab experiments would greatly streamline this process by filtering out potentially harmful peptides (Robles‐Loaiza et al., 2022). Such computational tools would enable the prioritization of the most promising candidates for further experimental validation.

Computational methods for predicting toxic peptides can be broadly categorized into two groups: conventional bioinformatics tools and machine learning (ML) models (Robles‐Loaiza et al., 2022). Conventional tools rely on similarity or homology‐based searches. For instance, BLAST (Altschul, 1997) and BLAST‐score (Altschul, 1997) classify a peptide sequence as toxic if it shares similarities with known toxic sequences, determined by a threshold applied to the E‐value. InterProScan (Quevillon et al., 2005) and HmmSearch (Potter et al., 2018), on the other hand, detect toxic peptide domains and categorize a sequence as toxic if it possesses or is associated with these domains. Since peptide sequences are typically short, searching for sequence similarity or domain matches in databases often does not yield sufficient information for the classification task. In contrast, ML models are trained to predict toxic peptides based on their specific characteristics. ML models have demonstrated notably higher predictive performance in toxicity assessment compared to conventional bioinformatics tools (Robles‐Loaiza et al., 2022).

ML‐based methods are optimized to learn discriminative features extracted from peptide sequences to distinguish between toxic and nontoxic peptides. Although various methods, such as ToxinPred (Gupta et al., 2013), HemoPI (Chaudhary et al., 2016), ClanTox (Naamati et al., 2009), and HAPPENN (Timmons & Hewage, 2020), have been in use for the task, they typically rely on handcrafted sequence‐derived or physicochemical features as inputs to their models. This limits their ability to explore the input data for potentially more informative features for toxicity prediction. Furthermore, these methods often overlook the inherent order present in input peptide sequences. Some existing methods, such as Toxify (Cole & Brewer, 2019), and ToxDL (Pan et al., 2021) have primarily been trained on long protein sequences and are not specifically designed for short peptide sequences. ATSE (Wei et al., 2021) incorporates position‐specific scoring matrices (PSSMs) and molecular graphs for toxicity prediction. However, generating PSSMs relies on the PSI–BLAST algorithm, which is both database‐dependent and time‐consuming to run (Wei et al., 2022). ToxIBTL (Wei et al., 2022) utilizes transfer learning by leveraging knowledge learned from protein toxicity for improved peptide toxicity prediction. Importantly, none of the cited methods above exploit the potential of the 3D structures of peptides, which can be a valuable source of information for toxicity prediction.

**FIGURE 1:**
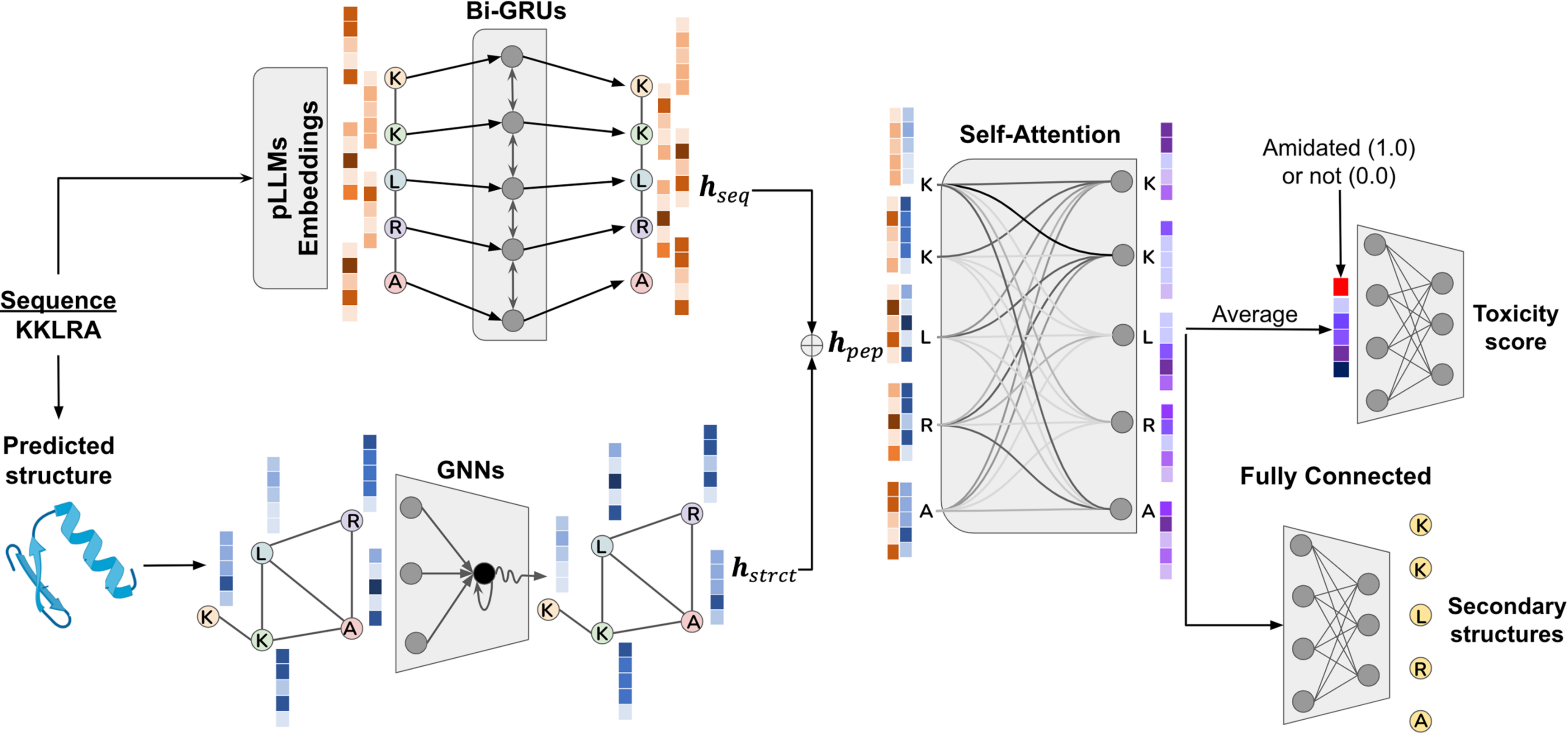
tAMPer model architecture. The orange vectors represent the initial sequence embeddings and sequential features obtained after applying bi‐GRUs. The blue vectors depict the initial graph‐encoded features and their transformation by GNNs. These sequential and structural features are combined and processed in a self‐attention layer, resulting in refined peptide representations, shown as purple vectors. Subsequently, these vectors are averaged, and a binary value (shown as a red index) indicating whether the peptide is amidated or not is appended. The augmented vectors are then passed through a fully connected layer to predict peptide toxicity. Additionally, the model performs secondary structure predictions for individual residues within the peptides.

Here, we introduce tAMPer, a novel multimodal structure‐aware deep learning model designed for predicting peptide toxicity. First, leveraging the capabilities of ESM2 (Lin et al., 2023) protein language model, we generate initial embeddings for peptide sequences, capturing higher‐level amino acid representations. We then incorporate ColabFold‐predicted 3D (Mirdita et al., 2022) structures represented as graphs, where amino acid residues and interactions between them, are nodes and edges, respectively. To learn from both sequence and graph representations, tAMPer utilizes three main components: (i) Bi‐directional Gated Recurrent Units (Bi‐GRUs) (Cho et al., 2014) for capturing sequential features, (ii) Graph Neural Networks (GNNs) (Scarselli et al., 2009) for extracting structural patterns from graph‐encoded structures, and (iii) self‐attention layer (Vaswani et al., 2017) for integrating sequential and structural features of each amino acid residue within peptides (Figure 1). Our model jointly learns from both sequential and structural data, and to our knowledge, it is the first ML model to do so for in silico toxicity prediction. Our contributions also include the creation of carefully curated training and validation sets of peptides with wet‐lab validated toxicity, which were used for training the tAMPer model. tAMPer holds promise for advancing the field of peptide toxicity prediction and may contribute to the development of safer and more effective AMPs.

## 2 RESULTS

### 2.1 In‐house peptide hemolysis dataset

**FIGURE 2:**
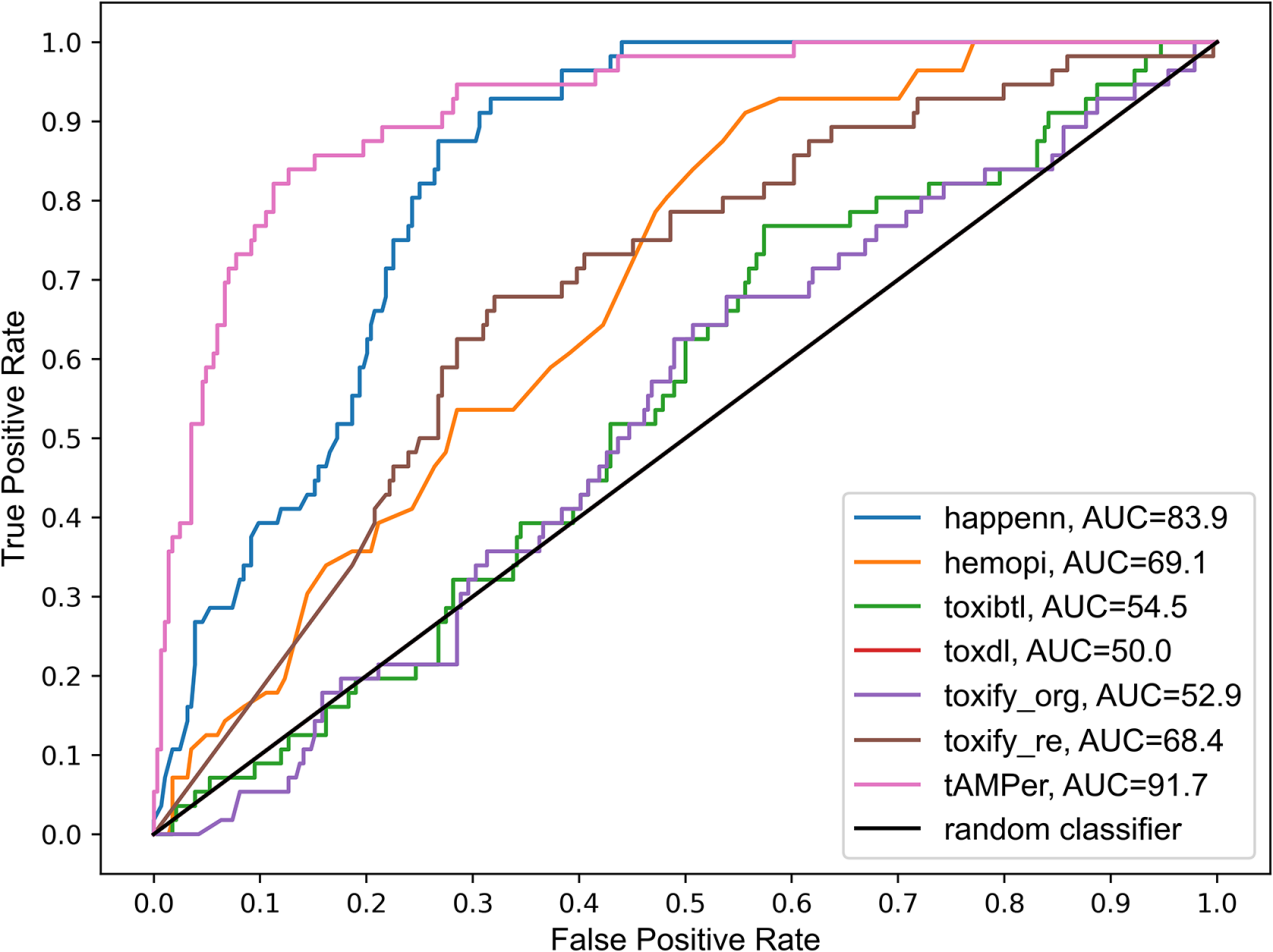
ROC curve comparing tAMPer and other toxicity prediction methods on the peptide hemolysis dataset. tAMPer is compared to the following methods: Toxify_org, which refers to the original Toxify method; toxify_re, which represents the re‐trained model of Toxify using our collected data; HAPPENN; HemoPI; ToxIBTL; and ToxDL. The ROC curve measures the true positive rate (sensitivity) against the false positive rate (1‐specificity) at different classification thresholds.

To assess the performance of tAMPer and other tools on real‐world data, we used an independent in‐house hemolysis test set consisting of 56 hemolytic (toxic) peptides and 284 nonhemolytic (nontoxic) peptides. Although the Toxify method allowed us to re‐train their model on our training dataset, none of the other methods provided this option. Therefore, we used the original models of these methods to predict toxicity and compared the results against tAMPer. For the ToxIBTL model, we obtained predictions from their online server (https://server.wei-group.net/ToxIBTL/Server.html). Similarly, predictions for the ToxinPred, ToxDL, HAPPENN, and HemoPI models were obtained using the default configurations from their respective online servers (http://crdd.osdd.net/raghava/toxinpred/, http://www.csbio.sjtu.edu.cn/bioinf/ToxDL/, https://research.timmons.eu/happenn, and https://webs.iiitd.edu.in/raghava/hemopi/). For Toxify, we downloaded the model from their GitHub repository (https://github.com/tijeco/toxify) and re‐trained on our collected training data using the default hyperparameters provided by the authors. The ATSE online server at http://server.malab.cn/ATSE was not functioning correctly when accessed in July 2023, so we were unable to obtain predictions from their model. As shown in Table 2, tAMPer outperforms the other methods, achieving an F1‐score of 68.7%, Matthews Correlation Coefficient (MCC) of 62.7%, area under the Receiver Operating Characteristic curve (auROC) of 91.7%, and area under the Precision‐Recall curve (auPRC) of 69.0%. Specifically, with tAMPer, we observed a 23.4% higher F1‐score, 23.3% in MCC, 7.8% in auROC, and 10.8% in auPRC over the second‐best method in each metric. Additionally, we analyzed the performance of all tools using Receiver Operating Characteristic (ROC) curves, which illustrate the true positive rate versus the false positive rate at different classification thresholds (Figure 2). Notably, tAMPer achieved the highest auROC 91.7%, indicating a higher discriminatory ability in distinguishing between toxic and nontoxic peptides.

#### 2.1.1 Correlation between tAMPer's toxicity probability and HC50

**FIGURE 3:**
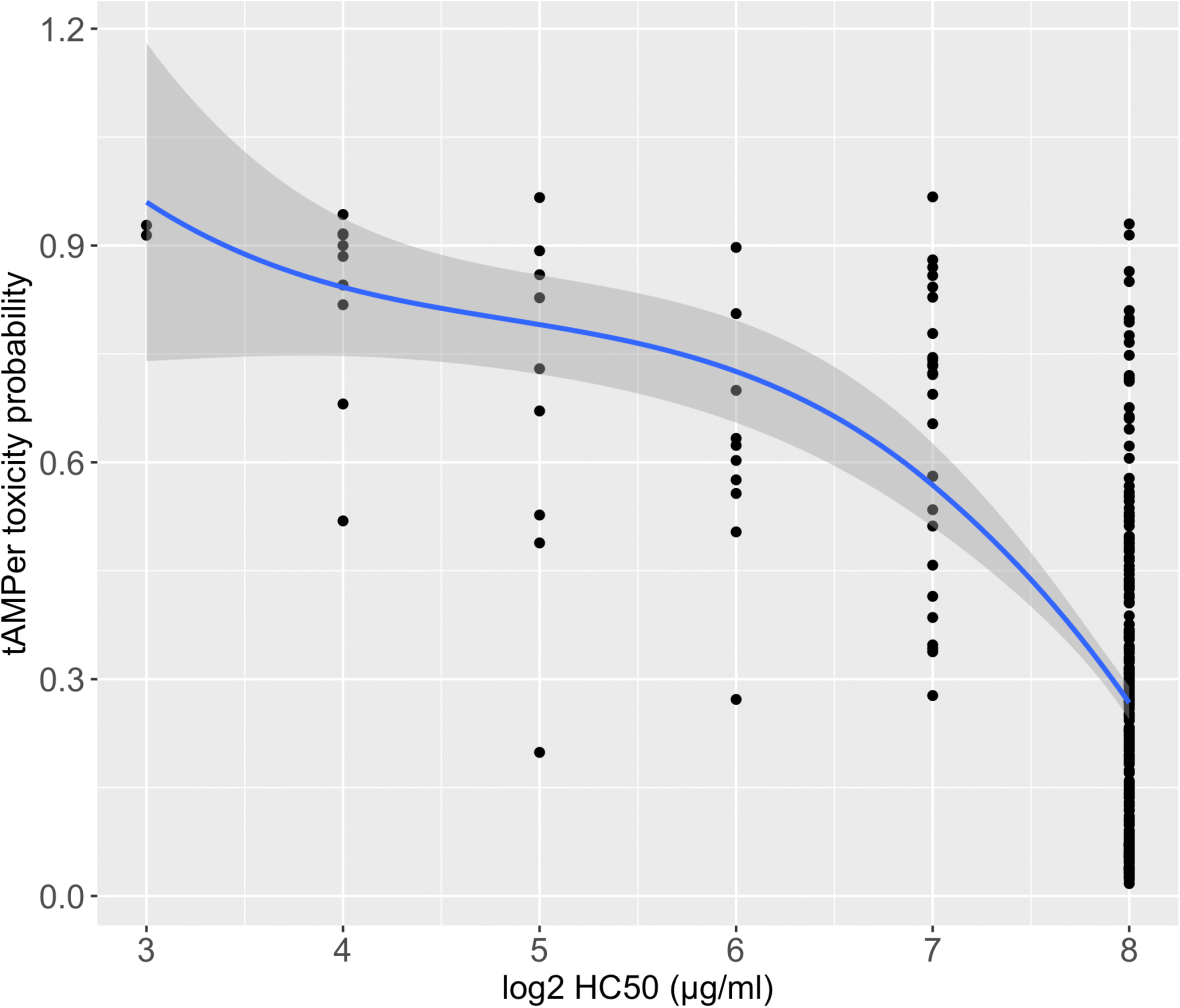
Correlation between tAMPer toxicity prediction probability and the measured HC50 values on the peptide hemolysis dataset. Each point on the graph corresponds to a specific peptide in the dataset. The y‐axis indicates the predicted toxicity probability metric output by tAMPer, whereas the *x*‐axis represents the corresponding logarithm (base 2) of the HC50 value expressed in μg/mL. The blue curve in the figure represents a locally fitted regression curve that captures the correlation between these two variables. The gray band surrounding the curve depicts the confidence interval, indicating the uncertainty associated with the fitted regression curve.

We examined the relationship between the toxicity probability predicted by tAMPer and logarithmic transformation (base 2) of the peptide concentration causing 50% hemolysis of red blood cells (HC50) measured in μg/mL (Figure 3). The observed pattern indicates an inverse correlation between the HC50 values and the predicted toxicity probabilities. As the HC50 values increase, indicating a higher concentration required for hemolysis, the predicted toxicity probabilities decrease, indicating a lower likelihood of peptide toxicity. As shown in Figure 3, the fitted curve exhibits a gradual decline in toxicity probability for peptides within the toxic HC50 range of 8 to 128 μg/mL, yet mostly remaining above a probability of 0.5. Meanwhile, peptides with HC50 values exceeding 128 μg/mL (nontoxic) display a probability of less than 0.3. It is worth noting that the model was not provided with HC50 values during the training process, highlighting the ability of tAMPer to infer the relationship between peptide toxicity and HC50 values without direct knowledge of these concentrations. This correlation aligns with the desired behavior of a reliable toxicity prediction model.

### 2.2 Protein toxicity benchmark

To ensure an unbiased evaluation of tAMPer and to avoid any potential biases from relying solely on our curated data, we conducted additional training and testing using the dataset established by the toxDL method (Pan et al., 2021). This benchmark dataset offers several advantages, including its widespread use by various methods in the literature. Thus, it serves as an impartial and objective evaluation of tAMPer's performance. The toxDL training set consists of 4472 toxic and 6341 nontoxic protein sequences, respectively. In the test set, each sequence has less than 40% sequence identity to any sequence in the training set. Moreover, to ensure diversity, none of the sequences in the training and test sets belong to the same Pfam clans.

In this study, and in line with other competitors, we evaluated the F1‐score, MCC, auROC, and auPRC. To maintain fairness in comparison with other competitors, we refrained from augmenting the data with supplementary structures (see Section 4 for details). Instead, we exclusively utilized the structure with the highest average per‐residue confidence score (pLDDT) for each sequence. We optimized the hyperparameters of tAMPer using the validation set (see Section 4 for details).

We compared tAMPer's performance against other methods evaluated on the protein benchmark dataset, which includes ToxDL and additional models referenced in the ToxDL paper. The methods that have been applied on this dataset include BLAST (Altschul, 1997), BLAST‐score (Altschul, 1997), InterProScan (Quevillon et al., 2005), HmmSearch (Potter et al., 2018), ClanTox (Naamati et al., 2009), ToxinPred (Gupta et al., 2013), ToxDL (Pan et al., 2021), and ToxIBTL (Wei et al., 2022). We re‐trained and tested Toxify (Cole & Brewer, 2019) on this dataset. Table 3 presents a comparison of tAMPer with these existing methods. tAMPer outperforms the competitors across all measured metrics, achieving an F1‐score of 86.0%, MCC of 85.0%, auROC of 99.2%, and auPRC of 91.6%. More specifically, tAMPer achieves a 3.0% higher F1‐score, 3.4% higher MCC, 0.3% higher auROC, and a 0.3% higher auPRC over the next best method on each metric (Table 3).

### 2.3 Ablation study

To assess the effectiveness of each component in our model, we conducted an ablation study. We isolated each source of information individually and compared the results against the performance of unaltered tAMPer, which combines both sources simultaneously. We created variations of the model as follows using the same architecture and parameters as they appear in tAMPer: (1) Sequence‐only model, which only utilizes the peptide sequence embeddings as input. The output of bi‐GRUs is passed to the self‐attention layer directly, disregarding the structural features (see Section 4 for details). (2) Structure‐only model, which only uses the three‐dimensional structural information of the peptides, neglecting the sequence information. All the tAMPer sub‐models were trained and tested on the peptide and protein toxicity datasets. The sequence‐only model performs better than the structure‐only model across all metrics in both datasets. On the protein benchmark, the sequence‐only model achieves 85.4% F1‐score, 84.2% MCC, 98.8% auROC, and 90.8% auPRC. However, through jointly learning from both sequential and structural information, tAMPer enhances performance to 86.0% F1‐score, 85.0% MCC, 99.2% auROC, and 91.6% auPRC, as presented in Table 2. On the in‐house hemolysis test set, tAMPer improves the performance of the sequence‐only sub‐model by 8.9% on Sensitivity, 10.2% on Specificity, 16.8% on F1‐score, 20.6% on MCC, 6.4% on auROC and 15.5% on auPRC, as shown in Table 3.

### 2.4 Optimal hyperparameters

In tAMPer, we introduce two hyperparameters, λ and $d_{\max}$, which respectively determine the weight balance between the two loss functions and the threshold for considering two residues to be in contact within the 3D structure (see Section 4 for details). On our peptide dataset, λ=0.1 or λ=0.2 consistently yield higher F1‐scores compared to other λs across varying $d_{\max}$ values, with the combination of λ=0.2 and $d_{\max}=12Å$ being as the optimal choice (Table S1). In the protein toxicity benchmark, we observed λ=0.4 and $d_{\max}=12Å$ performs the best according to the F1‐score.

## 3 DISCUSSION

tAMPer uses both sequential and structural data to predict the toxicity of peptides, as the functional attributes of peptides are assumed to originate not only from their amino acid composition but also from their structural characteristics (DeGrado et al., 1982; Fjell et al., 2012; Hollmann et al., 2016). We represent the input peptide using sequence and graph representations. The sequence representation aims to provide the sequential order of amino acid residues in peptides, whereas the graph representation captures spatial interactions between amino acid residues in the 3D structure as well as structural properties (see Section 4 for details). By having the two representations, tAMPer has access to a more comprehensive and informative representation of peptides.

Our model is built upon amino acid embeddings generated by ESM2 (Lin et al., 2023) and ColabFold (Jumper et al., 2021) predicted structures. The ESM2 embeddings offer several advantages over traditional encoding methods such as one‐hot encoding. Firstly, PLM‐based embeddings require fewer training examples compared to other methods, making them more efficient in data utilization. In addition, models using PLM‐based embeddings can be trained more quickly due to the pre‐trained nature of the input embeddings. Finally, PLM‐based embeddings have demonstrated generalizability in various protein‐related tasks, such as de novo protein design (Ferruz & Höcker, 2022; Lin et al., 2023) and structure prediction (Chowdhury et al., 2022; Lin et al., 2023). We used ColabFold for 3D structure prediction as it couples Mmseqs2 homology search with AF2 models, which enables a faster prediction time than AF2 (Mirdita et al., 2022).

To capture sequential features, tAMPer uses Bi‐GRUs, a lightweight recurrent neural network. This choice is motivated by the fact that the initial sequence embeddings are already high‐level representations derived from a PLM. For learning from structure‐encoded graphs, we utilized Geometric Vector Perceptrons (GVPs) (Jing et al., 2021), as they are 3D translation and rotation equivariant GNNs architecture designed to work specifically with 3D protein structures. To identify potential connections between amino acid residues that contribute to toxicity, tAMPer uses a self‐attention mechanism. The self‐attention layer computes attention weights (see Section 4 for details), providing transparency to the tAMPer's predictions. This allows for an assessment of which amino acid residues, or combinations thereof, have a substantial impact on determining whether a peptide is toxic or nontoxic (Figure S3).

To ensure a balanced integration of sequential and structural features, particularly considering the PLM‐generated nature of sequence embeddings, we took deliberate steps to ensure the contribution of structural features to predictions. We first pre‐trained the GNNs component of tAMPer on a reverse folding task, which is predicting amino acids from the 3D structure data (see Section 4 for details). Through this pre‐training approach, the model learns the intricate relationships between structural patterns and amino acid identities. We then exploit the inherent advantage of utilizing 3D structure‐derived attributes for predicting secondary structure, as opposed to only using sequential features (Figure S2). By adding secondary structure prediction loss to the overall loss function (see Section 4 for details), we ensure that the model learns from structural data during the training phase. The inclusion of this loss term not only allows the model to individually learn the secondary structure for each residue but also serves as a regularizer to prevent overfitting. We have shown that multi‐modal tAMPer outperforms its own sequence‐only and structure‐only sub‐models, and the optimal value of the λ hyperparameter $\left(\lambda >0\right)$ in both datasets indicates the importance of the secondary structure prediction. Our results indicate that in applications with longer sequences (>50 amino acids), tAMPer's performance can be improved by training with higher values of λ, as this can better capture structural features within larger 3D structures.

It is important to note that the class imbalance in our datasets accurately reflects their true distributions, captured in the collected data, protein toxicity benchmark, and our in vitro findings. We have observed a higher prevalence of nontoxic peptides/proteins compared to toxic ones. In assessing tAMPer's performance, we used metrics specifically designed to address class imbalance. These metrics include Sensitivity, Specificity, F1‐score, MCC, as well as auROC and auPRC.

tAMPer's performance on our independent peptide hemolysis test set, which is representative of real‐world experimental data, highlights its robustness. In the evaluation of sensitivity and specificity, certain methods exhibited a trade‐off between these metrics, leaning towards either excessively high sensitivity or specificity in their predictions. In contrast, tAMPer achieved the most balanced results overall, with 82.1% and 88.7% prediction sensitivity and specificity, respectively.

The toxicity of peptides can exhibit variation, and this variability is quantified by the HC50 value (Horváti et al., 2017). A balance often exists between a peptide's efficacy as an antimicrobial agent and its potential toxicity to host cells. In some cases, there is a trade‐off to consider, where peptides with high antimicrobial activity might also exhibit host cell toxicity. tAMPer's ability to predict the probability of toxicity provides valuable insights into this balance. The correlation between tAMPer's predicted probability of toxicity and the HC50 values of peptides facilitates the prioritization and selection of AMP candidates for subsequent experimental testing.

tAMPer's architecture and objective function can be adapted for predicting diverse protein/peptide functions. The ability to fine‐tune relative attributes on structure‐encoded graphs and the usage of various sequence embedding may be used to customize tAMPer for specific applications. To showcase this versatility, we extended tAMPer's application to a related task of identifying longer toxic proteins using a well‐established dataset.

tAMPer provides an in silico proxy for testing the hemolytic activity of peptides, primarily aimed at reducing the number of AMP candidates that need to be screened using costly wet lab experiments. Due to the potential disparity between in silico toxicity predictions and in vitro observations, subsequent in vitro investigations are required to validate the safety of identified AMPs. It is crucial to adjust tAMPer's threshold for toxicity classification to control false‐positive and false‐negative rates. If the goal is to maximize discovery, a higher tolerance for false negatives may be acceptable. Conversely, when faced with budget constraints and a large candidate pool, minimizing false positives becomes imperative. Expanding validation sets can aid in optimizing the threshold to achieve a better balance between false positives and negatives.

To predict toxicity, tAMPer utilizes five ColabFold‐predicted structures for a given peptide (see Section 4 for details), considering the variations in predicted structures and simulating different possible conformations of peptide structures. However, it is important to acknowledge the inherent limitations associated with using static 3D structure predictions. Proteins and peptides can adopt various conformations in different cellular environments, and a single static representation may not fully capture the dynamic nature of their function in a biological context (Fowler & Williamson, 2022; Mahlapuu et al., 2016). The predicted 3D structures used in tAMPer provide valuable insights into the potential structural characteristics of the peptides. However, these 3D structures might not be fully representative of the actual conformation of a peptide in a specific environment or when interacting with specific targets (McDonald et al., 2023). The ColabFold predictions are based on AF2 (Jumper et al., 2021), which, like any computational method, has its own assumptions and limitations. Notably, shorter protein and peptide sequences are under‐represented in AF2's training set. Future advancements in the field of computational protein structure prediction and refinement techniques will further improve our ability to capture the dynamic nature of peptides in biological environments and enable more accurate predictions of peptide functionality.

The performance of deep learning models often improves with an increase in the amount of available training data. However, in the field of toxicity prediction, it is important to acknowledge that the scale of the training data used is relatively limited compared to domains such as computer vision or natural language processing. There are several factors that contribute to the limited availability of training data for peptide toxicity prediction tasks. Firstly, experimental determination of peptide toxicity is a time‐consuming, costly, and labor‐intensive process. This leads to a scarcity of well‐annotated peptide datasets with reliable toxicity labels. Gathering comprehensive and high‐quality toxicity data requires significant resources and expertise. Furthermore, the mechanisms of toxicity and the complete toxicology profiles of peptides are still not fully understood (Fjell et al., 2012). Peptides can exhibit diverse modes of toxicity, and their toxicological properties can vary depending on the target organism or cell type (Greco et al., 2020). The lack of comprehensive knowledge about the mechanisms underlying peptide toxicity poses challenges in data collection and model development. As the field progresses and more research is conducted, more well‐annotated peptide sequence datasets will become available, and researchers will gain a deeper understanding of peptide toxicity mechanisms. This will contribute to improved model performance and a better understanding of peptide toxicity in various biological contexts.

We foresee tAMPer having wide‐ranging applications in predicting protein and peptide toxicity, encompassing, in particular, its relevance to AMPs, offering valuable insights for the development of more effective peptide‐based therapeutics to address the pressing challenge of antimicrobial resistance. Its ability to predict toxicity profiles with high accuracy holds the potential to streamline the screening and design of antimicrobial peptides, facilitating the discovery of novel candidates with improved safety profiles. By reducing the reliance on labor‐intensive and costly wet lab experiments, tAMPer has the potential to not only accelerate the discovery process but also to contribute to substantial cost savings.

## 4 MATERIALS AND METHODS

### 4.1 Data collection

#### 4.1.1 Training and validation sets

We compiled a dataset by aggregating relevant peptide sequences from several manually curated databases, including DBAASP v3 (Pirtskhalava et al., 2021), hemolytik (Gautam et al., 2014), APD3 (G. Wang et al., 2016), and UniProtKB/Swiss‐Prot (The UniProt Consortium et al., 2023) (accessed in Jan 2023). To ensure the quality and relevance of our dataset, we included peptide sequences that were 5–50 residues in length containing only natural amino acids, and filtered out redundant entries. Notably, we also incorporated peptide sequences with C‐terminal amidation, as this post‐translational modification can impact the toxicity of peptides (Fjell et al., 2012). We also accommodated this modification in our model.

From sequences obtained from the DBAASP (Pirtskhalava et al., 2021) and hemolytik (Gautam et al., 2014) databases, which provided experimental data on hemolytic activities, we used rigorous thresholds to classify them as either hemolytic (toxic) or nonhemolytic (nontoxic), as described in Table 1. Sequences that failed these criteria were excluded, resulting in 1604 toxic and 4042 nontoxic peptides. To supplement this dataset, we included all 104 nonredundant sequences from the APD3 database (Wang et al., 2016) with validated hemolytic activity as positive samples. From the Swiss‐Prot database (The UniProt Consortium et al., 2023), we downloaded sequences associated with the keyword “hemolysis.” We retained mature peptide sub‐sequences if the search results contained peptide annotations. If these annotations were not available, we considered the sub‐sequences that had been annotated as the main chain in the sequence. In cases where neither of these options was present, the entire sequence was included. We labeled the collected sequences as nontoxic if the function description included the following keywords: “No hemolysis,” “low hemolysis,” or “weak hemolysis,” and as toxic otherwise. These steps yielded 221 hemolytic and 5 nonhemolytic new sequences from the Swiss‐Prot database.

**TABLE 1:** Criteria for determining whether a peptide is hemolytic (toxic) or nonhemolytic (nontoxic).

Hemolytic peptides		Nonhemolytic peptides	
Hemolytic activity (%)	Concentration (μg/mL)	Hemolytic activity (%)	Concentration (μg/mL)
≥40	≤200	≤50	>250
≥50	≤250	≤40	>200
≥60	≤300	≤30	>150
≥70	≤350	≤20	>100
≥80	≤400	≤10	>50
≥90	≤450		
=100	≤500		

**TABLE 2:** Performance comparison of tAMPer, its sub‐models, and other methods on the in‐house peptide hemolysis dataset.

Method	Sensitivity	Specificity	F1‐score	MCC	auROC	auPRC
HAPPENN	**100.0**	52.5	45.3	39.2	83.9	43.3
HemoPI	92.9	33.5	35.0	21.5	69.1	25.5
ToxinPred	00.0	**98.6**	00.0	−04.8	‐	‐
ToxDL	26.8	65.9	17.9	−05.8	50.0	58.2
Toxify (original)	57.1	51.4	28.3	06.4	52.9	16.4
Toxify (re‐trained)	69.6	60.9	37.9	22.8	68.4	38.0
tAMPer (sequence‐only)	73.2	78.5	51.9	41.9	85.3	53.5
tAMPer (structure‐only)	63.5	64.3	36.7	20.9	72.5	45.2
tAMPer	82.1	88.7	**68.7**	**62.5**	**91.7**	**69.0**

**TABLE 3:** Performance comparison of tAMPer and other methods on the protein benchmark dataset.

Method	Category	F1‐score (%)	MCC (%)	auROC (%)	auPRC (%)
BLAST	Non‐ML	80.0	80.1	‐	‐
BLAST‐score	Non‐ML	78.9	77.5	86.8	81.8
InterProScan	Non‐ML	34.7	40.2	‐	‐
HmmSearch	Non‐ML	18.5	30.7	‐	‐
ClanTox	ML	62.0	60.4	90.3	61.2
ToxinPred‐RF	ML	66.7	63.8	94.8	71.6
ToxinPred‐SVM	ML	67.7	64.8	93.8	71.2
Toxify (original)	ML	71.5	69.0	93.0	74.3
Toxify (re‐trained)	ML	48.6 (±2.8)	45.0 (±3.3)	87.2 (±1.1)	52.4 (±2.4)
ToxDL	ML	80.9 (±2.2)	79.3 (±2.4)	98.9 (±0.2)	91.3 (±1.4)
ToxIBTL	ML	83.0 (±0.7)	81.6 (±0.8)	95.3 (±0.1)	84.7 (±0.2)
tAMPer (sequence‐only)	ML	**85.4 (±1.7)**	**84.2 (±1.8)**	**98.8 (±0.2)**	**90.8 (±1.1)**
tAMPer (structure‐only)	ML	**63.6 (±4.4)**	**60.8 (±4.7)**	**94.0 (±0.5)**	**67.9 (±5.1)**
tAMPer	ML	**86.0 (±1.8)**	**85.0 (±1.8)**	**99.2 (±0.0)**	**91.6 (±1.5)**

*Note*: To account for randomness during the training of deep learning models, the reported results for Toxify, ToxDL, ToxlBTL, and tAMPer represent the average outcomes across 10 independent runs, and the ranges indicate standard deviation. The highest value for each metric is bolded.

Our final nonredundant dataset consists of 1929 hemolytic and 4047 nonhemolytic peptide sequences. We split this dataset into training and validation sets with a ratio of 4:1 for each class, respectively. To ensure that the validation set was representative of the overall distribution of peptide sequences in the dataset and to remove the potential bias of learning sequence similarity, we used CD‐HIT (Fu et al., 2012) to select validation samples that share less than 80% sequence similarity with the training sequences. This approach helps to ensure that our model will generalize well to new peptide sequences. The datasets are available at https://github.com/bcgsc/tAMPer/tree/master/data.

#### 4.1.2 In‐house peptide hemolysis dataset

To comprehensively evaluate the performance of tAMPer and compare it with other existing methods, we created an independent set of 340 peptide sequences whose hemolytic activity we had assessed in vitro. The peptide sequences and their mutants were identified or designed by our previously published tools: AMPlify (Li et al., 2022), rAMPage (Lin et al., 2022), and AMPd‐Up (https://github.com/bcgsc/AMPd-Up). We obtained whole blood from healthy donor pigs (Lampire Biological Laboratories; Pipersville, PA, USA) and isolated the red blood cells (RBCs) through centrifugation and washing with Roswell Park Memorial Institute medium (RPMI; Thermo Fisher Scientific, MA, USA), to prepare a 1% solution (v/v) in RPMI. Lyophilized AMPs (GenScript; Piscataway, NJ, USA) were suspended and serially diluted in RPMI from 1280 down to 10 μg/mL in 96‐well polypropylene plates (Greiner Bio‐One; Kremsmünster, Austria) and combined with 100 μL of the 1% RBC solution. Following incubation at 37°C for 30–45 min, plates underwent centrifugation, and half of the supernatants were transferred to new 96‐well plates. Absorbance was measured at 415 nm utilizing the Cytation 5 Cell Imaging Multimode Reader (BioTek, CA, USA), with the AMP concentration causing 50% hemolysis of RBCs (HC50) serving as the hemolytic activity indicator. Absorbance readings from wells containing RBCs treated with 11 μL of a 2% Triton‐X100 solution and RPMI (AMP solvent‐only), established the baseline of 100% and 0% hemolysis, respectively. All centrifugation steps were done with the Allegra‐6R centrifuge (Beckman Coulter, CA, USA) at 500*g* for 5 min. To remove biases and confounding factors in experiments, each hemolysis assessment was performed in technical duplicates and repeated three times (*N* = 3). We labeled a peptide as toxic if it showed an HC50 value of less than or equal to 128 μg/mL in at least two experiments. This approach helped to ensure that the hemolytic peptides were consistently active over multiple trials and reduced the likelihood of false positives due to experimental variability. A description of our in‐house test set comprising 56 hemolytic and 284 nonhemolytic peptides is provided in Table S2, listing peptide names, sequences, and HC50 values.

#### 4.1.3 Three‐dimensional structure prediction

We utilized version 1.3.1 of local ColabFold (Mirdita et al., 2022) to predict the 3D structures of peptides. To run ColabFold locally, we obtained the model inputs, including multiple sequence alignments and structural templates, from ColabFold's server. We generated five structures corresponding to the five sub‐models, each initialized using a different random seed. The output structures are ranked based on their average pLDDT score, which represents the per‐residue confidence of ColabFold in its predictions.

#### 4.1.4 Data augmentation

To augment our collected data, we not only selected the structure with the highest average pLDDT score as is customary but also considered the other four structures. We used all five structures as separate samples during the training process. When evaluating a given peptide at the test time, tAMPer predicts the toxicity probability for each of the five structures and then calculates the final output as the average probability across these five predicted structures.

### 4.2 Evaluation metrics

We evaluated tAMPer using sensitivity, specificity, F1‐score, Matthews Correlation Coefficient (MCC), area under the Receiver Operating Characteristic curve (auROC), and area under the Precision‐Recall curve (auPRC). These metrics were calculated as follows:

$$\text{Sensitivity}=\frac{\mathrm{TP}}{\mathrm{TP}+\mathrm{FN}},$$

$$\text{Specificity}=\frac{\mathrm{TN}}{\mathrm{TN}+\mathrm{FP}},$$

$$F1-\text{score}=\frac{\mathrm{TP}}{\mathrm{TP}+\frac{1}{2}\left(\mathrm{FP}+\mathrm{FN}\right)},$$

(1) $$\mathrm{MCC}=\frac{\mathrm{TP}\times \mathrm{TN}-\mathrm{FP}\times \mathrm{FN}}{\sqrt{\left(\mathrm{TP}+\mathrm{FP}\right)\times \left(\mathrm{TP}+\mathrm{FN}\right)\times \left(\mathrm{TN}+\mathrm{FP}\right)\times \left(\mathrm{TN}+\mathrm{FN}\right)}},$$

where TP, TN, FP, and FN are true‐positive, true‐negative, false‐positive and false‐negative values, respectively. Sensitivity and specificity assess the model's ability to correctly identify true positives and true negatives, whereas the F1‐score, MCC, auROC, and auPRC provide an evaluation of the overall performance.

### 4.3 tAMPer model

#### 4.3.1 Encoding peptide sequences

To represent peptide sequences numerically, we obtain sequence embeddings from PLMs trained on millions of raw protein sequences. In this particular work, the ESM2 (Lin et al., 2023) PLM is utilized to generate initial embeddings for amino acids in peptides. For each peptide sequence, the embedding is represented as $X=[x_{1}$, $x_{2}$, …, 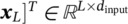, where $x_{i}$ corresponds to a vector representation of the amino acid residue at position i, L is the maximum sequence length (default = 50) and $d_{\text{input}}$ is the dimensionality of the input embeddings obtained from ESM2. If the input sequences are shorter than the maximum length, X is zero‐padded on the right.

#### 4.3.2 Sequence processing module

Bi‐GRUs in tAMPer capture the sequential dependencies between the amino acid residues in peptide sequences. Bi‐GRUs are able to do this by maintaining a hidden state that represents the “memory” of the network, allowing it to encode and retain information about previous residues as it processes the current residue. The bi‐directional nature of the Bi‐GRUs allows for processing the sequence both forwards and backward, enabling it to capture dependencies in both directions. Bi‐GRUs transform the input embeddings $X\in {\mathbb{R}}^{L\times d_{\text{input}}}$ to $h_{\mathrm{seq}}\in {\mathbb{R}}^{L\times d_{h}}$, representing the extracted sequential features for each amino acid in model's dimensionality $d_{h}$. For the forward direction and residue at sequence position i, the feature vector $h_{\mathrm{seq}}^{\left(i\right)}$ is computed as a function of the previous hidden state $h_{\mathrm{seq}}^{\left(i-1\right)}$ and the current input embedding $x_{i}$, using a learnable function $f_{W}$ parameterized by a weight matrix W:

(2) $$h_{\mathrm{seq}}^{\left(i\right)}=f_{W}\;\left(h_{\mathrm{seq}}^{\left(i-1\right)},x_{t}\right),$$

for 1<i≤L. The reverse direction would work in a similar way, replacing $h_{\mathrm{seq}}^{\left(i-1\right)}$ with $h_{\mathrm{seq}}^{\left(i+1\right)}$ for 1≤i<L. Bi‐GRUs formulates $f_{W}$ based on reset and update gates, determining how much information should be forgotten and updated in each step, respectively (Figure S1).

#### 4.3.3 Encoding 3D structures as graphs

In tAMPer, we represent the 3D structure of a peptide as a graph $G\left(V,E\right)$, where V is the set of residues and E is the set of edges encoding the interactions between residues. To determine whether two residues should be connected by an edge, we evaluate the distance between their $C_{\alpha }$ atoms in 3D space, and if this distance is less than a certain threshold $d_{\max}$, we establish an edge between the residues. This criterion indicates that the two amino acids are in contact or in close proximity to each other.

#### 4.3.4 Structure processing module

We utilize GNNs to capture structural patterns in graph‐encoded peptides. GNNs operate based on a message‐passing paradigm, which includes three steps: featurization, aggregation, and updating of node representations (Battaglia et al., 2018). During featurization, the relevant numerical features are encoded on the nodes and edges of the graphs. In the aggregation step, each node collects information from its neighboring edges and nodes. Finally, each node updates its feature vector based on received messages from the aggregation step and its previous state. This process of aggregation and updating can be repeated multiple times through layers of GNNs to iteratively refine the node representations.

For each node $n_{i}\in V$ and edge $e_{\mathrm{ij}}\in E$, with i and j represent two positions in the peptide sequence, we utilize backbone structural properties to initialize hidden feature vectors $h_{\text{strct}}^{\left(i\right)}$ and $h_{\text{strct}}^{\left(i,j\right)}$ for nodes and edges, respectively, similar to Jing et al. (2021). Both $h_{\text{strct}}^{\left(i\right)}$ and $h_{\text{strct}}^{\left(i,j\right)}$ are functions of scaling ($s_{∙}$) and vector ($v_{∙}$) features, as in $h_{\text{strct}}^{\left(i\right)}=\left(s_{i},v_{i}\right)$ and $h_{\text{strct}}^{\left(i,j\right)}=\left(s_{i,j},v_{i,j}\right)$, whose components are defined as follows.

The initial $s_{i}$ for each residue is a vector consisting of sine and cosine values of the dihedral angles ω,ψ and φ. Let $C_{\alpha_{i}}$ and $C_{\beta_{i}}$ be the spatial position of the alpha‐ and beta‐carbon in the *i*‐th amino acid in a peptide sequence, respectively. We initialize the feature vector$\;v_{i}$ by concatenating the following three vectors: (i) the forward unit vector in the direction of $r_{i}=C_{\alpha_{i+1}}-C_{\alpha_{i}}$, $\forall i\in \left[1,L\right)$; (ii) the backward unit vector in the direction of $b_{i}=C_{\alpha_{i-1}}-C_{\alpha_{i}}$, $\forall i\in \left(1,L\right]$; and (iii) a unit vector in the estimated direction of $d_{i}=C_{\beta_{i}}-C_{\alpha_{i}}$. The last forward ($r_{L}$) and first backward ($b_{1}$) vectors are initialized to zero vector. We initialize $s_{i,j}$ with the distance between $C_{\alpha_{j}}$ and $C_{\alpha_{i}}$ in 3D space using 16 Gaussian radial basis functions (Equation S1), and sinusoidal encoding of distance along the backbone j−i (Equation S2). We establish $v_{i,j}$ as the unit vector in the direction from $C_{\alpha_{j}}$ to $C_{\alpha_{i}}$. Note that $v_{i,j}=-v_{j,i}$ as the direction of the vectors is opposite.

During the aggregation step, every node $n_{i}$ in the graph computes the messages received from its neighboring nodes $n_{j}$ and edges in between, $e_{\mathrm{ij}}$, using a learnable function $f_{\mathrm{msg}}$. These messages are then averaged to obtain.

(3) $$m_{i}=\frac{1}{N_{i}}\sum_{j:e_{i,j}\in E}f_{\mathrm{msg}}\left(h_{\text{strct}}^{\left(i\right)},h_{\text{strct}}^{\left(j\right)},h_{\text{strct}}^{\left(i,j\right)}\;\right),$$

where $N_{i}$ is the total number of received messages for $n_{i}$.

Then, each node updates its hidden feature vector $h_{\text{strct}}^{\left(i\right)}$ using its current state and the aggregated messages $m_{i}$ through a learnable function $f_{\text{update}}$.

(4) $$h_{\text{strct}}^{\left(i\right)}\leftarrow f_{\text{update}}\left(h_{\text{strct}}^{\left(i\right)},m_{i}\right).$$

The learnable functions $f_{\mathrm{msg}}$ and $f_{\text{update}}$ are parametrized neural networks. We formulated the aggregation (Equation 2) and updating (Equation 3) steps using Geometric Vector Perceptrons (GVPs) (Jing et al., 2021) with layer normalizations similar to Jing et al. (2021) as follows,

(5) $$m_{i}=\text{LayerNorm}\left(h_{\text{strct}}^{\left(i\right)}+\frac{1}{N_{i}}\;\sum_{j:e_{i,j}\in E}f\left(h_{\text{strct}}^{\left(j\right)}\;\|\;h_{\text{strct}}^{\left(i,j\right)}\right)\right),$$

(6) $$h_{\text{strct}}^{\left(i\right)}\leftarrow \text{LayerNorm}\left(h_{\text{strct}}^{\left(i\right)}+g\left(m_{i}\right)\right),$$

where || sign indicates the concatenation of two vectors, f and g are sequences of two and three layers of GVPs, respectively.

We pre‐trained the GNNs component on a reverse‐folding task with the predicted structures data as well as structures of sequences with less than 100 amino acids length available from AlphaFold2 database (Varadi et al., 2022). The objective is to predict the corresponding amino acid type for each node within featured graphs. Each node is assigned a label from the set $\left\{a_{1},a_{2},\ldots ,a_{20}\right\}$, representing the specific natural amino acid it corresponds to. Throughout the training process, the model utilizes the initial scalar and vector features present in the graphs as inputs to predict these labels. Given that the features do not explicitly convey information about the amino acids themselves, GNNs rely on the structural features and the conformation of the graph to predict the amino acids.

#### 4.3.5 Integration of sequential and structural features

The initial representation of peptides is formed by concatenating the extracted sequential and structural features for each amino acid residue i obtained from Bi‐GRUs and GNNs as follows,

(7) $${\forall i\in \left[1,L\right]:h}_{\mathrm{seq}}^{\left(i\right)}={\text{Dropout}}_{2D}\left(h_{\mathrm{seq}}^{\left(i\right)}\right),$$

(8) $${\forall i\in \left[1,L\right]:h}_{\text{strct}}^{\left(i\right)}={\text{Dropout}}_{2D}\left(h_{\text{strct}}^{\left(i\right)}\right),$$

(9) $$h_{\mathrm{pep}}={\left[h_{\mathrm{seq}}^{\left(1\right)}\|h_{\text{strct}}^{\left(1\right)},.\ldots ,h_{\mathrm{seq}}^{\left(L\right)}\|h_{\text{strct}}^{\left(L\right)}\right]}^{T}\in {\mathbb{R}}^{L\times 2d_{h}}.$$

To avoid overreliance on a single source of information during training, a two‐dimensional dropout with p=0.5 probability was applied separately to the sequential and structural features before their concatenation.

tAMPer utilizes an 8‐headed self‐attention layer (Vaswani et al., 2017) to combine the sequential and structural features in a same space. To compute the attention on i‐th residue within each attention head, query $q_{i}={\mathrm{Qh}}_{\mathrm{pep}}^{\left(i\right)}$, key $k_{i}=Kh_{\mathrm{pep}}^{\left(i\right)}$, and value $v_{i}=Vh_{\mathrm{pep}}^{\left(i\right)}$ matrices with learnable weights $Q,K,V\in {\mathbb{R}}^{d_{a}\times d_{h}}$ are constructed, where $d_{a}=\frac{d_{h}}{8}$. Then, for the *i*‐th residue, the attention weights $a_{\mathrm{ij}}$ and a refined representation ${h\prime }_{\mathrm{pep}}^{\left(i\right)}$ are computed as,

(10) $$a_{\mathrm{ij}}=\frac{\exp\left(q_{i}^{T}k_{j}\right)}{\sum_{t=1}^{L}\exp\left(q_{i}^{T}k_{t}\right)},$$

(11) $${h\prime }_{\mathrm{pep}}^{\left(i\right)}=\sum_{j=1}^{L}a_{\mathrm{ij}}v_{j},$$

as suggested by Bahdanau et al. (2015). For toxicity prediction, the final representations of peptides is obtained as,

(12) $${\bar{h^{\prime }}}_{\mathrm{pep}}=\text{Dropout}\left(\frac{1}{L}\;\sum_{i=1}^{L}{h^{\prime }}_{\mathrm{pep}}^{\left(i\right)}\right).$$

A single binary feature (AMD) is appended to indicate whether the peptide is amidated or not, ${\left[{\bar{h^{\prime }}}_{\mathrm{pep}},\mathrm{AMD}\right]}^{T}$. This augmented vector is then fed into a fully connected layer, followed by a softmax activation function. The softmax function outputs a probability representing the predicted likelihood of toxicity. We classify the input peptide as “toxic,” if the output probability is greater than 0.5, and as “nontoxic” otherwise. We use the Binary Cross Entropy loss function to calculate the error between the predicted probability and the true label of toxicity, denoted as $L_{\mathrm{tx}}$.

We also introduce an additional loss term, denoted as $L_{\mathrm{ss}}$, which utilizes the combined features $h\prime_{\mathrm{pep}}$ to predict the secondary structure of each amino acid individually. To predict the secondary structure, one layer of fully connected network is used to map the combined features $h\prime_{\mathrm{pep}}\in {\mathbb{R}}^{L\times 2d_{h}}$ to a dimension of ${\mathbb{R}}^{L\times 8}$, where 8 represents the number of classes of secondary structures. The cross‐entropy loss function is then applied to calculate the error between the predicted secondary structure and the true labels. The final loss is a linear combination of the toxicity loss $L_{\mathrm{tx}}$ and the secondary structure loss $L_{\mathrm{ss}}$, with a hyperparameter 0≤λ≤1 controlling the weighting between the two losses:

(13) $$L=\left(1-\lambda \right)L_{\mathrm{tx}}+\lambda L_{\mathrm{ss}}.$$

We determining the optimal value for λ through hyperparameter tuning on the validation set.

#### 4.3.6 Implementation details

The tAMPer model was implemented using PyTorch (Paszke et al., 2019) version 1.13.1 along with PyTorch Geometric library (Fey & Lenssen, 2019) version 2.3.0. For the peptide hemolysis dataset, we utilized a hidden dimension size of $d_{h}=64$, one layer of GNNs, and one layer of bi‐GRUs. We generated amino acid embeddings for each sequence using the t12 variant (Lin et al., 2023) of the ESM2 model. To accommodate the longer sequences and larger structures in the protein toxicity benchmark, we utilized t33 embeddings from the ESM2 model, increased the number of GNN layers to 3, and augmented the dimensionality of the model to 128.

We applied layer normalization after each main component of the model, namely bi‐GRUs, GNNs, and self‐attention layer. The Adam (Kingma & Ba, 2015) optimizer with a batch size of 32 was used to optimize the loss function and update the weights of tAMPer. The initial learning rate was set to $4\times 10^{-4}$, and a weight decay of $10^{-7}$ was applied to prevent overfitting. In addition, a dropout rate of 0.5 was applied to the fully connected layer of the model for toxicity prediction. To determine the stopping point of the training process, we implemented an early stopping strategy. If the best F1‐score was not increasing on the validation set for 20 consecutive epochs, the training process would be stopped. The F1‐score was monitored due to the imbalanced number of positive and negative samples in the validation set.

### 4.4 Hyperparameter tuning

We determined the optimal values for hyperparameters, λ and $d_{\max}$, by jointly optimizing their performance on the validation sets of both datasets. We varied λ in range of $\left\{0.0,0.1,\ldots ,0.5\right\}$, where a value of 0.0 indicates no secondary structure prediction, and 0.5 assigns equal importance to secondary structure and toxicity prediction losses. In addition, we experimented with the different values for $d_{\max}$, including $\left\{8,10,12,20\right\}$ angstroms.

## AUTHOR CONTRIBUTIONS

**Hossein Ebrahimikondori:** Conceptualization; methodology; validation; formal analysis; software; data curation; visualization; writing – original draft; investigation; writing – review and editing. **Darcy Sutherland:** Validation; methodology; data curation; writing – review and editing; formal analysis. **Anat Yanai:** Methodology; validation; data curation; writing – review and editing; formal analysis. **Amelia Richter:** Methodology; validation; data curation; writing – review and editing; formal analysis. **Ali Salehi:** Methodology; validation; data curation; writing – review and editing; formal analysis. **Chenkai Li:** Methodology; formal analysis; writing – review and editing. **Lauren Coombe:** Writing – review and editing; formal analysis; software. **Monica Kotkoff:** Resources; project administration; writing – review and editing. **René L. Warren:** Formal analysis; writing – review and editing; supervision; methodology. **Inanc Birol:** Supervision; methodology; formal analysis; writing – review and editing; conceptualization; investigation; funding acquisition; resources.

## FUNDING INFORMATION

This work was supported by Genome BC and Genome Canada (291PEP). Additional support was provided by the Canadian Agricultural Partnership, a federal‐provincial‐territorial initiative, under the Canada‐BC Agri‐Innovation Program. The program is delivered by the Investment Agriculture Foundation of BC (INV106). Opinions expressed in this document are those of the authors and not necessarily those of the Governments of Canada and British Columbia or the Investment Agriculture Foundation of BC. The Governments of Canada and British Columbia, and the Investment Agriculture Foundation of BC, and their directors, agents, employees, or contractors will not be liable for any claims, damages, or losses of any kind whatsoever arising out of the use of, or reliance upon, this information.

## CONFLICT OF INTEREST STATEMENT

IB is a co‐founder of and executive at Amphoraxe Life Sciences Inc.

The values are reported as percentages. Highest value for each metric is bolded.

## Supporting information


**Data S1.** Supporting information.

**Data S2.** Supporting information.

## Data Availability

The datasets, code, and models can be accessed at https://github.com/bcgsc/tAMPer.
