# Supplementary material for: Structure‐aware deep learning model for peptide toxicity prediction
Source: Protein Sci. 2024 Jun 22;33(7):e5076. doi: 10.1002/pro.5076 (PMC11193153; doi:10.1002/pro.5076)
Supplement: Supplementary file 1 — Data S1. Supporting information. [file PRO-33-e5076-s002.docx]

**Supplementary Materials**

**Structure-aware deep learning model for peptide toxicity prediction**

Hossein Ebrahimikondori^1,2^, Darcy Sutherland^1,3,4^, Anat Yanai^1,3^, Amelia Richter^1,3^, Ali Salehi^1,3^, Chenkai Li^1,2^, Lauren Coombe^1^, Monica Kotkoff^1^, René L. Warren^1^, and Inanc Birol^1,3,4,5, *^

^1^ Canada’s Michael Smith Genome Sciences Centre, BC Cancer Agency, Vancouver, BC, V5Z 4S6, Canada

^2^ Bioinformatics Graduate Program, University of British Columbia, Vancouver, BC, V6T 1Z4, Canada

^3^ Public Health Laboratory, British Columbia Centre for Disease Control, Vancouver, BC, V5Z 4R4, Canada

^4^ Department of Pathology and Laboratory Medicine, University of British Columbia, Vancouver, BC, V6T 1Z4, Canada

^5^ Department of Medical Genetics, University of British Columbia, Vancouver, BC, V6H 3N1, Canada

* Correspondence: Inanc Birol (ibirol@bcgsc.ca)


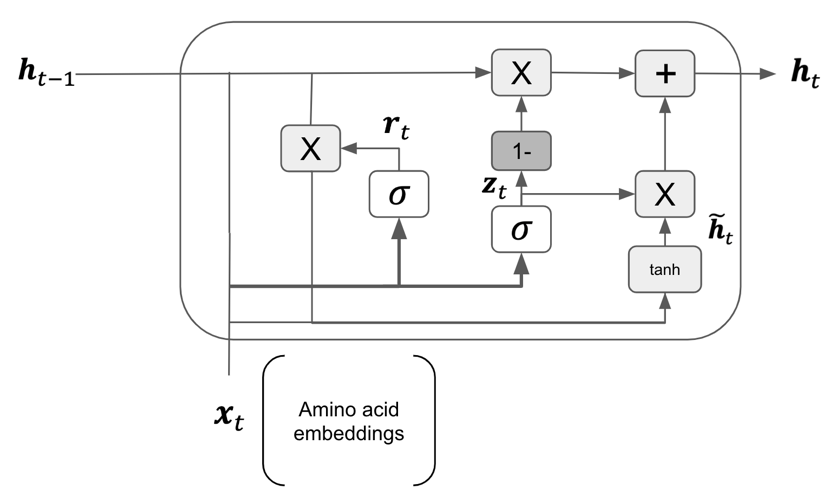


**Supplementary Figure 1**. schematic diagram of GRUs (Chung, Gulcehre, Cho, & Bengio, 2014).

For the $t$-th residue in a sequence, the $j$-th unit in the hidden vector $\boldsymbol{h}_{t}^{(j)}$ is computed as a linear combination of the $j$-th previous hidden vector in the sequence $\boldsymbol{h}_{t-1}^{(j)}$and the candidate vector ${\tilde{\boldsymbol{h}}}_{t}^{(j)}$:

$$\boldsymbol{h}_{t}^{(j)}=\left( 1-\boldsymbol{z}_{t}^{\left( j \right)} \right)\boldsymbol{h}_{t-1}^{\left( j \right)}+ \boldsymbol{z}_{t}^{\left( j \right)}{\tilde{\boldsymbol{h}}}_{t}^{(j)}$$

as described in (Chung et al., 2014). The update gate $\boldsymbol{z}_{t}^{\left( j \right)}$ decides how much the content $\boldsymbol{h}_{t}^{(j)}$ should be updated. The update gate is computed as follows,

$$\boldsymbol{z}_{t}^{\left( j \right)}=\sigma\left( \boldsymbol{W}_{z}\boldsymbol{x}_{t}+\boldsymbol{U}_{z}\boldsymbol{h}_{t-1} \right)^{\left( j \right)},$$

Where $\sigma$ indicates sigmoid function, $\boldsymbol{W}_{z}$ and $\boldsymbol{U}_{z}$ are learnable weight matrices, and $\boldsymbol{x}_{t}$ is the given $t$-th residue’s input embeddings. The candidate vector ${\tilde{\boldsymbol{h}}}_{t}^{(j)}$ is computed based on the input and how much should we forget from the previously computed hidden state.

$${\tilde{\boldsymbol{h}}}_{t}^{(j)}=\tanh\left( \boldsymbol{W}\boldsymbol{x}_{t}+\boldsymbol{U}(\boldsymbol{r}_{t}\odot\boldsymbol{h}_{t-1}) \right)^{\left( j \right)}$$

where $\boldsymbol{r}_{t}$ is a vector of reset gates for each unit and $\odot$ indicates element-wise multiplication. Each reset gate $\boldsymbol{r}_{t}^{\left( j \right)}$ is computed similarly to upadate gate as follows:

$$\boldsymbol{r}_{t}^{\left( j \right)}=\sigma\left( \boldsymbol{W}_{r}\boldsymbol{x}_{t}+\boldsymbol{U}_{r}\boldsymbol{h}_{t-1} \right)^{\left( j \right)}$$

**Supplementary equations 1**. **Gaussian radial basis functions**

$$\mu_{i}=d_{min}+i\times\frac{d_{max}-d_{min}}{count}$$

$$\sigma=\frac{d_{max}-d_{min}}{count}$$

$${rbf}_{i}\left( d \right)=\mathrm{ex}p \left\{ -\left( \frac{d-\mu_{i}}{\sigma} \right)^{2} \right\}$$

We used 16 Gaussian radial basis functions ($count=16$) with $d_{min}=0.0$ and $d_{max}=20$ Angstroms.

**Supplementary equations 2**. **sinusoidal encoding**

$$PE\left( distance, 2i \right)=\sin\left( \frac{distance}{{10000}^{\frac{2i}{d}}} \right)$$

$$PE\left( distance, 2i+1 \right)=\cos\left( \frac{distance}{{10000}^{\frac{2i}{d}}} \right)$$

Where $d$ ($=16$) is the number of embeddings and $i\in[0,16)$ indicates the $i$-th dimension.


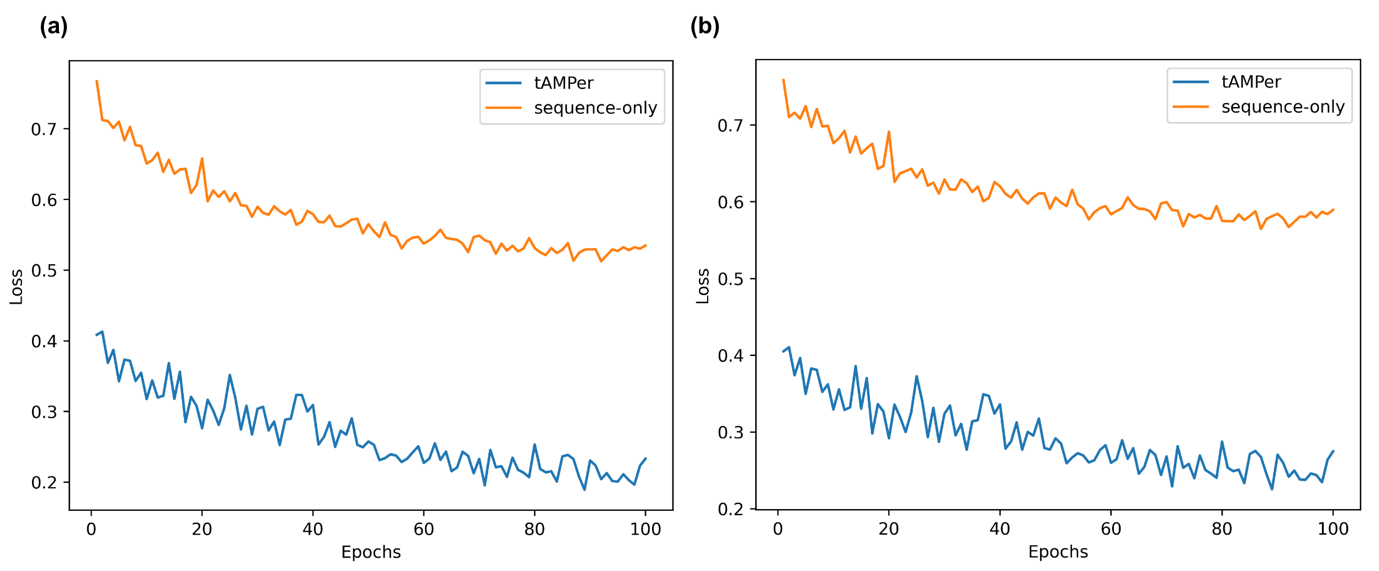


**Supplementary Figure 2**. **(a) Training and (b) validation loss curves for secondary structure prediction using tAMPer and sequence-only variant.** The orange curves represent the model's performance when utilizing only sequential features, while the blue curves depict the model's performance when employing both sequential and structural features. The training error for the sequence-only model remains relatively high even after 100 epochs, suggesting its difficulty in capturing relevant features for the given task, as anticipated. However, the results indicate that the structural features are highly effective in accurately predicting the secondary structure for each residue in the peptide sequences. Adding $\mathcal{L}_{ss}$ in tAMPer's loss function compels the model to include the extracted structural features as a component of the abstract representation of amino acids.
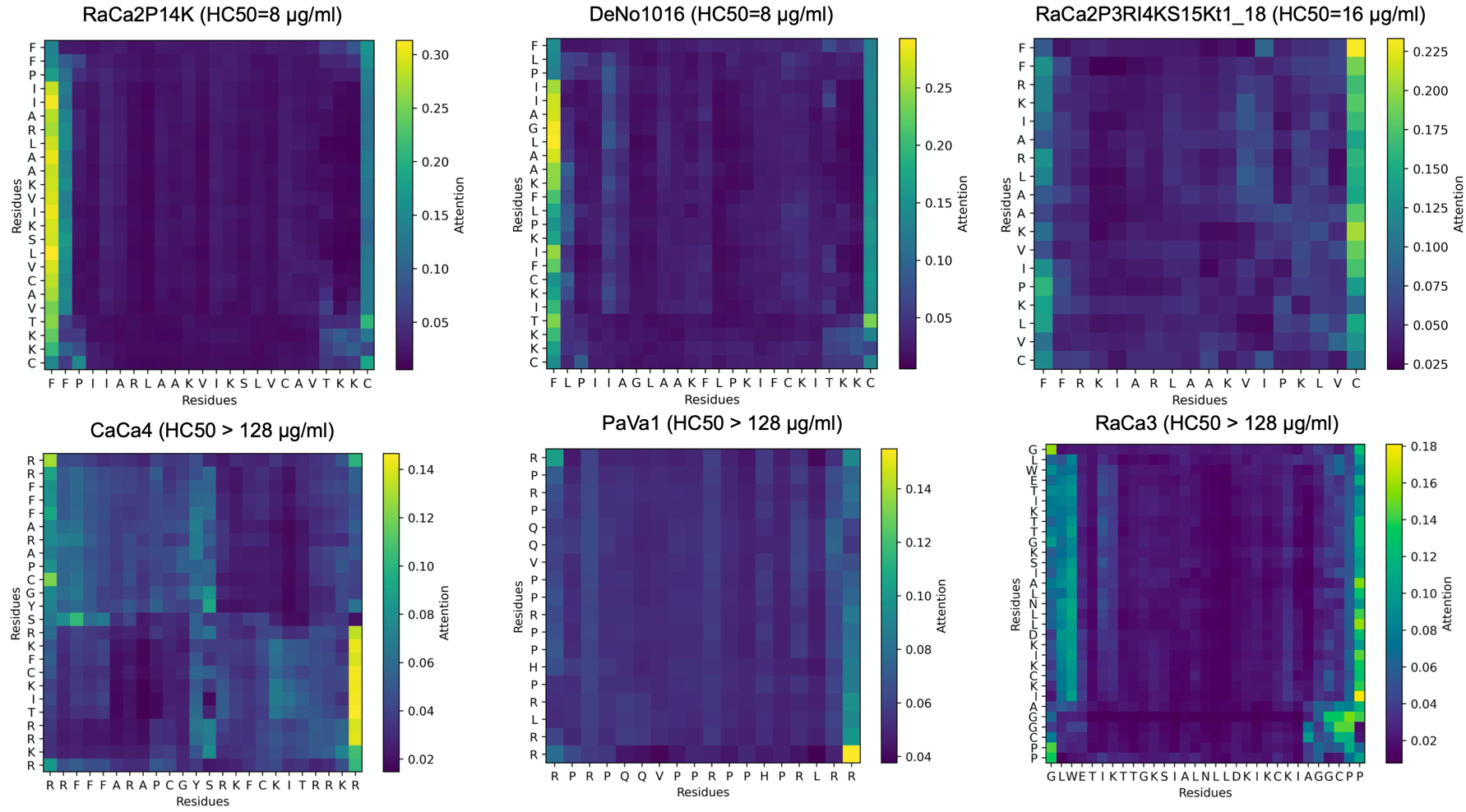
**Supplementary Figure 3**. **tAMPer’s attention maps.** The sequential and structural features are combined and processed in the self-attention layer, where each residue in the sequence attends to the other residues. For every prediction, tAMPer generates an attention map illustrating the attention distribution for each residue (rows) across the sequence (columns). The top row in the figure showcases the generated maps for the most toxic sequences in our in-house hemolysis test set, while the bottom row displays attention maps for three randomly selected sequences with minimal toxicity.

**Supplementary Table 1**. **Performance comparison of tAMPer based on different values of** $\boldsymbol{\lambda}$ **and** $\boldsymbol{d}_{\boldsymbol{max}}$ **on the validation dataset.** The metrics are presented as percentages. Highest value for each metric is bolded.

| $d_{max}$ | $\lambda$ | **Sensitivity** | **Specificity** | **F1** | **MCC** | **auROC** | **auPRC** |
| --- | --- | --- | --- | --- | --- | --- | --- |
| 8 | 0.0 | 68.9 | 64.7 | 55.8 | 31.1 | 71.4 | 50.3 |
|  | 0.1 | 70.8 | 68.6 | 58.9 | 36.8 | 74.0 | 54.9 |
|  | 0.2 | 61.2 | 75.8 | 57.0 | 35.7 | 73.9 | 57.2 |
|  | 0.3 | 65.9 | 71.3 | 57.5 | 35.1 | 73.8 | 54.4 |
|  | 0.4 | 65.9 | 70.1 | 56.8 | 33.8 | 72.4 | 52.8 |
|  | 0.5 | 65.6 | 68.9 | 55.9 | 32.3 | 72.2 | 52.4 |
| 10 | 0.0 | 67.5 | 68.2 | 56.8 | 33.4 | 72.7 | 52.6 |
|  | 0.1 | 63.7 | 72.7 | 56.8 | 34.6 | 73.0 | 52.7 |
|  | 0.2 | 63.7 | 75.9 | 58.7 | 38.1 | **75.1** | 56.4 |
|  | 0.3 | 54.1 | 76.9 | 52.7 | 30.6 | 71.7 | 50.5 |
|  | 0.4 | 60.1 | 75.6 | 56.1 | 34.5 | 73.6 | **57.3** |
|  | 0.5 | 60.1 | 73.6 | 55.0 | 32.3 | 72.7 | 51.9 |
| 12 | 0.0 | 59.6 | 74.3 | 55.1 | 32.6 | 72.4 | 54.0 |
|  | 0.1 | 63.9 | 73.4 | 57.4 | 35.6 | 74.0 | 53.0 |
|  | 0.2 | 67.5 | 73.3 | **59.6** | **38.7** | 74.9 | 55.7 |
|  | 0.3 | 58.2 | 73.4 | 53.7 | 30.4 | 71.1 | 49.2 |
|  | 0.4 | 56.8 | 74.9 | 53.5 | 30.8 | 72.1 | 52.5 |
|  | 0.5 | 68.9 | 65.1 | 56.0 | 31.6 | 72.2 | 50.6 |
| 20 | 0.0 | 52.7 | **82.8** | 55.3 | 36.6 | 72.6 | 53.2 |
|  | 0.1 | 66.7 | 70.1 | 57.3 | 34.6 | 73.0 | 55.5 |
|  | 0.2 | **71.0** | 65.3 | 57.3 | 33.7 | 72.7 | 51.4 |
|  | 0.3 | 63.1 | 73.2 | 56.8 | 34.6 | 74.0 | 53.6 |
|  | 0.4 | 66.4 | 70.0 | 57.0 | 34.2 | 73.8 | 53.1 |
|  | 0.5 | 54.4 | 81.7 | 55.8 | 36.6 | 74.8 | 55.0 |

**Supplementary Table 2. Peptide hemolysis dataset.** Each hemolysis assessment was performed in triplicate (N=3) to determine the concentration required for 50% hemolysis of red blood cells (HC50). A peptide is labeled toxic if it exhibits an HC50 value of less than or equal to 128 µg/ml in at least two technical replicates.

| **Peptide name** | **Sequence** | **HC50 (µg/ml)** | | | **Label** |
| --- | --- | --- | --- | --- | --- |
|  |  | **N=1** | **N=2** | **N=3** |  |
| OdMa12 | GFMDTAKNVAKNVAVTLLYNLKCKITKAC | >128 | >128 | >128 | non-toxic |
| PeNi7 | VIPFVASVAAEMMHHVYCAASKRCKN | >128 | >128 | >128 | non-toxic |
| PeNi10 | GLLLDTVKGAAKNVAGILLNKLKCKVTGDC | >128 | >128 | >128 | non-toxic |
| PeNi11 | GILTDTLKGAAKNVAGVLLDKLKCKITGGC | >128 | >128 | >128 | non-toxic |
| PeNi14 | GLWTTIKEGVKNFSVGVLDKIRCKITGGC | >128 | >128 | >128 | non-toxic |
| PeNi16 | ATAWKVPPGLQPIRPIRIRPLCGNDKS | >128 | >128 | >128 | non-toxic |
| RaOm5 | AGYSRMIRRPPGFSPFRVAPASSLKR | >128 | >128 | >128 | non-toxic |
| RaSy2 | EEQRFLPVVAGLAAKVLPSIICAVTKKC | >128 | >128 | >128 | non-toxic |
| BoAr6 | GILRLVTRRFRFSPTNLNRYTVARLVSGVP | >128 | >128 | >128 | non-toxic |
| TeRu3 | AVLSFVHKLFLNFLHVDTSKGKCRATLQ | >128 | >128 | >128 | non-toxic |
| TeRu4 | SWLSKSVKKLVNKKNYTRLEKLAKKKLFNE | >128 | >128 | >128 | non-toxic |
| PaVa2 | KYHHIKLRHGRHRRTIH | >128 | >128 | >128 | non-toxic |
| PaVa3 | ITEPVGTKAPTFTSELRGGWLKKR | >128 | >128 | >128 | non-toxic |
| PaVi1 | WALRWKTR | >128 | >128 | >128 | non-toxic |
| PoRo1 | VAAFAIIGCLCCRRPRR | >128 | >128 | >128 | non-toxic |
| PoSn2 | TALKSLSILKKLAKLNM | >128 | >128 | >128 | non-toxic |
| TeRu1 | VPFGLKPR | >128 | >128 | >128 | non-toxic |
| TeRu2 | AFVRILCYCCPRRIKRR | >128 | >128 | >128 | non-toxic |
| VeSi1 | FILHAKKTRSAK | >128 | >128 | >128 | non-toxic |
| OdMa13 | GFMDTAKNVAKNVAVTLLDNLKCKITKAC | >128 | >128 | >128 | non-toxic |
| OdTo4 | KLMIPRKKRGIFGGLLKVGKKIACGLSGLC | >128 | >128 | >128 | non-toxic |
| PeNi8 | GILLNTLKGAAKNVAGVLLDKLKCKITGGC | >128 | >128 | >128 | non-toxic |
| LiVe1 | GWLDIAKKVASVVAGIVKR | >128 | >128 | >128 | non-toxic |
| LiVe2 | GWLDIAKKVASVVAGLGKR | >128 | >128 | >128 | non-toxic |
| OdMa1 | GLLSGILGAGKKIVCGFSGLC | >128 | >128 | >128 | non-toxic |
| OdMa3 | GLLSGLLGAGKKIVCGLSGMC | >128 | >128 | >128 | non-toxic |
| OdMa4 | GILSGLLGAGKKIVC | >128 | >128 | >128 | non-toxic |
| OdMa6 | GLLSGVLGVGKKIVCGLSGLC | >128 | >128 | >128 | non-toxic |
| OdMa9 | GLISGILGAGKKVLC | >128 | >128 | >128 | non-toxic |
| OdMa10 | GLISGILGAGKKVLCGLSGLC | >128 | >128 | >128 | non-toxic |
| OdTo1 | GILSGLLGAGKKLACGLIGLC | ≥128 | >128 | >128 | non-toxic |
| OdTo2 | GIFGGHLKVGKKIACGLSGLC | >128 | >128 | >128 | non-toxic |
| OdTo3 | GIFGGLLKEGKKIACGLSGLC | >128 | >128 | >128 | non-toxic |
| PeNi2 | GLLGKVLGVGKKVLCVVSGLC | >128 | >128 | >128 | non-toxic |
| PeNi3 | GIFSLIKGAAKVVAKGLG | >128 | >128 | >128 | non-toxic |
| PeNi4 | GLLGKVLGVGKKVLC | >128 | >128 | >128 | non-toxic |
| PeNi5 | GLLGKVLGVGKKVLCGVTGRERCQ | >128 | >128 | >128 | non-toxic |
| RaOm2 | GILSGLLGAGKKIVCGLSGMC | >128 | >128 | >128 | non-toxic |
| RaOm3 | GIFSLIKGAAKVVAKGLGK | >128 | >128 | >128 | non-toxic |
| RaOm4 | GLLGKVLGVGKKVLCGVSGRC | >128 | >128 | >128 | non-toxic |
| RaSi1 | GLVGKLVKGGLKLIGHVANG | >128 | >128 | >128 | non-toxic |
| AnFl2 | GILRSLGWIQMPRSRRRHR | >128 | >128 | >128 | non-toxic |
| ApCe1 | GIYTGRLLPVYIPQPRPPHPRLRR | >128 | >128 | >128 | non-toxic |
| BoUs1 | RKIIAVSVHKLCRVKR | >128 | >128 | >128 | non-toxic |
| CaCa1 | FACPIGFFRLKR | >128 | >128 | >128 | non-toxic |
| CaCa2 | FIKTQVLKHLVAGVRVARGLDWKWR | >128 | >128 | >128 | non-toxic |
| CaCa4 | RRFFFATAPCGYSRKFCKITRRKR | >128 | >128 | >128 | non-toxic |
| DiLo1 | GAFVLWGPTPRPRRR | >128 | >128 | >128 | non-toxic |
| MyGu1 | RRAIFASIRGYLGLRKR | >128 | >128 | >128 | non-toxic |
| NaVi3 | KLFLTLWKLKR | >128 | >128 | >128 | non-toxic |
| PaVa1 | RPRPQQVPPRPPHPRLRR | >128 | >128 | >128 | non-toxic |
| CLIB_denovo9 | TLPDVAKGAAKGLAKTALDVLKCKLKGVC | >128 | >128 | >128 | non-toxic |
| CLIB_denovo10 | VLGSLLKGLGKLLGKILGKIGKKIGKCVGKC | 128 | 128 | >128 | toxic |
| CLIB_denovo19 | GLLSLLKKLLKKLC | >128 | 128 | 64 | toxic |
| CLIB_denovo14 | GMLSKIKGALKKVAKKILKKL | >128 | >128 | >128 | non-toxic |
| CLIB_denovo16 | GTLSKLLKKLFKKILKKL | ≥128 | 128 | 128 | toxic |
| CLIB_denovo11 | GVLSTLKKKLKKLLKKLLKTLLKKI | 128 | 64 | 32-64 | toxic |
| CLIB_denovo12 | RFGSIIKNVGKNVLKTLLCKIKKKC | >128 | >128 | 128 | non-toxic |
| CLIB_denovo13 | GWLSKLKKKGKKIGKAIKKIGKKF | >128 | >128 | >128 | non-toxic |
| CLIB_deno38 | TLPSLLSGLLKKL | >128 | >128 | >128 | non-toxic |
| CLIB_denovo26 | GDLSKLKGKGKKIGGKLLGGLKKKIKGVC | >128 | >128 | >128 | non-toxic |
| CLIB_denovo27 | LVGKLLKKLGKKIKKIF | >128 | >128 | >128 | non-toxic |
| CLIB_denovo17 | GWLSKLKKKGKKIGKAIKKIGKKFC | >128 | >128 | >128 | non-toxic |
| CLIB_denovo29 | WDWLKKKGKGVAGLVAKALKAALKAL | 128 | 128 | 128 | toxic |
| CLIB_denovo18 | GMLSKIKGALKKVAKKILKKLC | ≥128 | ≥128 | >128 | toxic |
| CLIB_denovo20 | GTLSKLLKKLFKKILKKLC | 64 | 128 | 128 | toxic |
| CLIB_denovo30 | DVLDTLKNAAGKLAKKLLKKLLKKI | >128 | >128 | >128 | non-toxic |
| RaCa3 | GLWETIKTTGKSIALNLLDKIKCKIAGGCPP | >128 | >128 | 128 | non-toxic |
| RaCa3T5K | GLWEKIKTTGKSIALNLLDKIKCKIAGGCPP | >128 | >128 | >128 | non-toxic |
| RaCa3T8K | GLWETIKKTGKSIALNLLDKIKCKIAGGCPP | 128 | 128 | ≥128 | toxic |
| RaCa3S12K | GLWETIKTTGKKIALNLLDKIKCKIAGGCPP | >128 | >128 | 64-128 | non-toxic |
| RaCa3A26R | GLWETIKTTGKSIALNLLDKIKCKIRGGCPP | >128 | >128 | >128 | non-toxic |
| RaCa3G28K | GLWETIKTTGKSIALNLLDKIKCKIAGKCPP | >128 | >128 | >128 | non-toxic |
| RaCa3P31K | GLWETIKTTGKSIALNLLDKIKCKIAGGCPK | >128 | >128 | 128 | non-toxic |
| RaCa3T5KT8K | GLWEKIKKTGKSIALNLLDKIKCKIAGGCPP | >128 | >128 | 128 | non-toxic |
| RaCa3T8KS12K | GLWETIKKTGKKIALNLLDKIKCKIAGGCPP | ≥128 | >128 | >128 | non-toxic |
| RaCa3S12KG28K | GLWETIKTTGKKIALNLLDKIKCKIAGKCPP | >128 | >128 | >128 | non-toxic |
| RaCa3S12KP31K | GLWETIKTTGKKIALNLLDKIKCKIAGGCPK | ≥128 | >128 | >128 | non-toxic |
| RaCa3T8KT9K | GLWETIKKKGKSIALNLLDKIKCKIAGGCPP | >128 | >128 | >128 | non-toxic |
| RaCa3T8KA26R | GLWETIKKTGKSIALNLLDKIKCKIRGGCPP | >128 | >128 | >128 | non-toxic |
| RaCa3G28KT8K | GLWETIKKTGKSIALNLLDKIKCKIAGKCPP | >128 | >128 | >128 | non-toxic |
| RaCa3T8KT9KS12K | GLWETIKKKGKKIALNLLDKIKCKIAGGCPP | >128 | >128 | >128 | non-toxic |
| RaCa3G28KT8KS12K | GLWETIKKTGKKIALNLLDKIKCKIAGKCPP | ≥128 | >128 | ≥128 | toxic |
| RaCa3T5KT8KG28K | GLWEKIKKTGKSIALNLLDKIKCKIAGKCPP | >128 | >128 | >128 | non-toxic |
| RaCa3T8KT9KG28K | GLWETIKKKGKSIALNLLDKIKCKIAGKCPP | >128 | >128 | >128 | non-toxic |
| RaCa3G27KT8KT9K | GLWETIKKKGKSIALNLLDKIKCKIAKGCPP | >128 | >128 | >128 | non-toxic |
| RaCa3T8KT9KG27KG28K | GLWETIKKKGKSIALNLLDKIKCKIAKKCPP | >128 | >128 | >128 | non-toxic |
| RaCa3T5KT8KT9KS12KG27KG28K | GLWEKIKKKGKKIALNLLDKIKCKIAKKCPP | >128 | >128 | >128 | non-toxic |
| RaCa3S12KC23SC29S | GLWETIKTTGKKIALNLLDKIKSKIAGGSPP | >128 | >128 | >128 | non-toxic |
| RaCa3t1_29 | GLWETIKTTGKSIALNLLDKIKCKIAGGC | >128 | >128 | >128 | non-toxic |
| RaCa3S12Kt1_29 | GLWETIKTTGKKIALNLLDKIKCKIAGGC | 128 | >128 | >128 | non-toxic |
| RaCa3T8KT9KG27KG28Kt1_29 | GLWETIKKKGKSIALNLLDKIKCKIAKKC | ≥128 | >128 | >128 | non-toxic |
| RaCa3T5KT8KT9KS12KG27KG28Kt1_29 | GLWEKIKKKGKKIALNLLDKIKCKIAKKC | >128 | >128 | >128 | non-toxic |
| RaCa3T5KT8KS12Kt1_29 | GLWEKIKKTGKKIALNLLDKIKCKIAGGC | >128 | >128 | >128 | non-toxic |
| RaCa3C29St1_29 | GLWETIKTTGKSIALNLLDKIKCKIAGGS | >128 | >128 | >128 | non-toxic |
| RaCa3S12Kt1_23 | GLWETIKTTGKKIALNLLDKIKC | ≥128 | >128 | ≥128 | toxic |
| RaCa3T5KT8Kt1_23 | GLWEKIKKTGKSIALNLLDKIKC | >128 | >128 | >128 | non-toxic |
| RaCa3T5KT8KS12Kt1_23 | GLWEKIKKTGKKIALNLLDKIKC | 128 | >128 | >128 | non-toxic |
| RaCa3T5KT8KC23St1_23 | GLWEKIKKTGKSIALNLLDKIKS | >128 | >128 | >128 | non-toxic |
| RaCa3S12KC23SKt1_23 | GLWETIKTTGKKIALNLLDKIKS | >128 | >128 | >128 | non-toxic |
| RaCa3T5KT8KS12C23SKt1_23 | GLWEKIKKTGKKIALNLLDKIKS | >128 | >128 | >128 | non-toxic |
| RaCa7 | FFPRVLPLANKFLPTIYCALPKSVGN | >128 | >128 | >128 | non-toxic |
| RaCa7P7K | FFPRVLKLANKFLPTIYCALPKSVGN | 64 | 32-64 | 64 | toxic |
| RaCa7P7R | FFPRVLRLANKFLPTIYCALPKSVGN | 64 | 64 | 64 | toxic |
| RaCa7N10K | FFPRVLPLAKKFLPTIYCALPKSVGN | >128 | >128 | >128 | non-toxic |
| RaCa7T15K | FFPRVLPLANKFLPKIYCALPKSVGN | >128 | >128 | >128 | non-toxic |
| RaCa7Y17K | FFPRVLPLANKFLPTIKCALPKSVGN | >128 | >128 | >128 | non-toxic |
| RaCa7S23R | FFPRVLPLANKFLPTIYCALPKRVGN | >128 | >128 | >128 | non-toxic |
| RaCa7G25R | FFPRVLPLANKFLPTIYCALPKSVRN | >128 | >128 | >128 | non-toxic |
| RaCa7Y17KT15K | FFPRVLPLANKFLPKIKCALPKSVGN | >128 | >128 | >128 | non-toxic |
| RaCa7Y17KP7K | FFPRVLKLANKFLPTIKCALPKSVGN | >128 | >128 | ≥128 | non-toxic |
| RaCa7Y17KP14K | FFPRVLPLANKFLKTIKCALPKSVGN | >128 | >128 | >128 | non-toxic |
| RaCa7P7RY17K | FFPRVLRLANKFLPTIKCALPKSVGN | >128 | >128 | >128 | non-toxic |
| RaCa7P7KG25R | FFPRVLKLANKFLPTIYCALPKSVRN | 32-64 | 32 | 32 | toxic |
| RaCa7L8KG25R | FFPRVLPKANKFLPTIYCALPKSVRN | >128 | >128 | >128 | non-toxic |
| RaCa7P7KN10K | FFPRVLKLAKKFLPTIYCALPKSVGN | 64-128 | 64 | 64 | toxic |
| RaCa7P7RT15K | FFPRVLRLANKFLPKIYCALPKSVGN | 64 | 32 | 64 | toxic |
| RaCa7Y17KP7KT15K | FFPRVLKLANKFLPKIKCALPKSVGN | >128 | >128 | >128 | non-toxic |
| RaCa7Y17KP14KT15K | FFPRVLPLANKFLKKIKCALPKSVGN | >128 | >128 | >128 | non-toxic |
| RaCa7P7RY17KT15K | FFPRVLRLANKFLPKIKCALPKSVGN | >128 | 128 | >128 | non-toxic |
| RaCa7P7KG25RR4K | FFPKVLKLANKFLPTIYCALPKSVRN | 64 | 32 | 128 | toxic |
| RaCa7L8KG25RR4K | FFPKVLPKANKFLPTIYCALPKSVRN | >128 | >128 | >128 | non-toxic |
| RaCa7P7KN10KY17K | FFPRVLKLAKKFLPTIKCALPKSVGN | 128 | 128 | ≥128 | toxic |
| RaCa7t1_18 | FFPRVLPLANKFLPTIYC | >128 | >128 | >128 | non-toxic |
| RaCa7Y17KT15Kt1_18 | FFPRVLPLANKFLPKIKC | >128 | >128 | >128 | non-toxic |
| RaCa7Y17KP7Kt1_18 | FFPRVLKLANKFLPTIKC | >128 | >128 | >128 | non-toxic |
| RaCa7Y17KP14Kt1_18 | FFPRVLPLANKFLKTIKC | >128 | >128 | >128 | non-toxic |
| RaCa7P7RY17Kt1_18 | FFPRVLRLANKFLPTIKC | 128 | 128 | >128 | toxic |
| RaCa7P7KG25Rt1_18 | FFPRVLKLANKFLPTIYC | >128 | >128 | >128 | non-toxic |
| RaCa7L8KG25Rt1_18 | FFPRVLPKANKFLPTIYC | >128 | >128 | >128 | non-toxic |
| RaCa7P7KN10Kt1_18 | FFPRVLKLAKKFLPTIYC | 16 | 32 | 32-64 | toxic |
| RaCa7P7RT15Kt1_18 | FFPRVLRLANKFLPKIYC | 32 | 32 | 64 | toxic |
| RaCa7Y17KP7KT15Kt1_18 | FFPRVLKLANKFLPKIKC | 128 | >128 | >128 | non-toxic |
| RaCa7Y17KP14KT15Kt1_18 | FFPRVLPLANKFLKKIKC | >128 | >128 | >128 | non-toxic |
| RaCa7P7RY17KT15Kt1_18 | FFPRVLRLANKFLPKIKC | 128 | 128 | >128 | toxic |
| RaCa7P7KG25RR4Kt1_18 | FFPKVLKLANKFLPTIYC | >128 | >128 | >128 | non-toxic |
| RaCa7L8KG25RR4Kt1_18 | FFPKVLPKANKFLPTIYC | >128 | >128 | >128 | non-toxic |
| RaCa7P7KN10KY17Kt1_18 | FFPRVLKLAKKFLPTIKC | 64 | 64 | 64 | toxic |
| RaCa7P7RT15KY17KP14Kt1_18 | FFPRVLRLANKFLKKIKC | 32 | 16 | 16 | toxic |
| RaCa7P7RT15K1Y17KN10Rt1_18 | FFPRVLRLARKFLPKIKC | 64 | 16 | 64 | toxic |
| RaCa7P7RT15KY17KP3Rt1_18 | FFRRVLRLANKFLPKIKC | 16-32 | 16 | 16 | toxic |
| RaCa7P7RT15KC18St1_18 | FFPRVLRLANKFLPKIYS | >128 | >128 | >128 | non-toxic |
| RaCa7Y17KP14KC18St1_18 | FFPRVLPLANKFLKTIKS | >128 | >128 | >128 | non-toxic |
| RaCa7Y17KP14KT15KC18St1_18 | FFPRVLPLANKFLKKIKS | >128 | >128 | >128 | non-toxic |
| RaCa7P7RY17KT15KC18St1_18 | FFPRVLRLANKFLPKIKS | >128 | >128 | >128 | non-toxic |
| Ranatuerin4 | FLPFIARLAAKVFPSIICSVTKKC | 16 | 16 | 16 | toxic |
| RaCa1 | GLLDIIKTTGKDFAVKILDNLKCKLAGGCPP | >128 | >128 | >128 | non-toxic |
| RaCa1I5K | GLLDKIKTTGKDFAVKILDNLKCKLAGGCPP | >128 | >128 | >128 | non-toxic |
| RaCa1T8K | GLLDIIKKTGKDFAVKILDNLKCKLAGGCPP | >128 | >128 | >128 | non-toxic |
| RaCa1D12K | GLLDIIKTTGKKFAVKILDNLKCKLAGGCPP | >128 | >128 | >128 | non-toxic |
| RaCa1P31K | GLLDIIKTTGKDFAVKILDNLKCKLAGGCPK | >128 | >128 | >128 | non-toxic |
| RaCa1P31R | GLLDIIKTTGKDFAVKILDNLKCKLAGGCPR | >128 | >128 | >128 | non-toxic |
| RaCa1T8KD12K | GLLDIIKKTGKKFAVKILDNLKCKLAGGCPP | >128 | >128 | >128 | non-toxic |
| RaCa1D12KP31K | GLLDIIKTTGKKFAVKILDNLKCKLAGGCPK | >128 | >128 | >128 | non-toxic |
| RaCa1T8KG27K | GLLDIIKKTGKDFAVKILDNLKCKLAKGCPP | >128 | >128 | >128 | non-toxic |
| RaCa1T8KI5K | GLLDKIKKTGKDFAVKILDNLKCKLAGGCPP | >128 | >128 | >128 | non-toxic |
| RaCa1T8KN20K | GLLDIIKKTGKDFAVKILDKLKCKLAGGCPP | >128 | >128 | >128 | non-toxic |
| RaCa1P30RP31R | GLLDIIKTTGKDFAVKILDNLKCKLAGGCRR | >128 | >128 | >128 | non-toxic |
| RaCa1T8KI5KD12K | GLLDKIKKTGKKFAVKILDNLKCKLAGGCPP | >128 | >128 | >128 | non-toxic |
| RaCa1T8KI5KG27K | GLLDKIKKTGKDFAVKILDNLKCKLAKGCPP | >128 | >128 | >128 | non-toxic |
| RaCa1T8KN20KD19K | GLLDIIKKTGKDFAVKILKKLKCKLAGGCPP | ≥128 | >128 | >128 | non-toxic |
| RaCa1T8KN20KG27K | GLLDIIKKTGKDFAVKILDKLKCKLAKGCPP | >128 | >128 | >128 | non-toxic |
| RaCa1T8KI5KD12KP30RP31R | GLLDKIKKTGKKFAVKILDNLKCKLAGGCRR | >128 | >128 | >128 | non-toxic |
| RaCa1T8KN20KD19KP30RP31R | GLLDIIKKTGKDFAVKILKKLKCKLAGGCRR | ≥128 | >128 | >128 | non-toxic |
| RaCa1t1_29 | GLLDIIKTTGKDFAVKILDNLKCKLAGGC | >128 | >128 | >128 | non-toxic |
| RaCa1T8KN20KD19Kt1_29 | GLLDIIKKTGKDFAVKILKKLKCKLAGGC | 128 | 128 | >128 | toxic |
| RaCa1C29St1_29 | GLLDIIKTTGKDFAVKILDNLKCKLAGGS | >128 | >128 | >128 | non-toxic |
| RaCa1D12Kt1_23 | GLLDIIKTTGKKFAVKILDNLKC | >128 | >128 | >128 | non-toxic |
| RaCa1D12KD19Kt1_23 | GLLDIIKTTGKKFAVKILKNLKC | ≥128 | >128 | >128 | non-toxic |
| RaCa1T8KN20KD19Kt1_23 | GLLDIIKKTGKDFAVKILKKLKC | >128 | >128 | >128 | non-toxic |
| RaCa1C23St1_23 | GLLDIIKTTGKDFAVKILDNLKS | >128 | >128 | >128 | non-toxic |
| RaCa1T8KN20KD19KC23St1_23 | GLLDIIKKTGKDFAVKILKKLKS | >128 | >128 | >128 | non-toxic |
| LeBo1 | GIFSLIKGAAK | >128 | >128 | >128 | non-toxic |
| OdMa8 | GLISGILGAGKK | >128 | >128 | >128 | non-toxic |
| PeNi6 | AGLQFPVGRIHRHLKTR | >128 | >128 | >128 | non-toxic |
| PeNi12 | GAPKGCWTKSYPPKPCSGKR | >128 | >128 | >128 | non-toxic |
| PeNi13 | KEERGAPKGCWTKSYPPKPCSGKR | >128 | >128 | >128 | non-toxic |
| PeNi15 | FLPSSPWNEGTYVLKKLKS | >128 | >128 | >128 | non-toxic |
| PeNi17 | RMIRRPPGFSPFRVAPASSLKR | >128 | >128 | >128 | non-toxic |
| PeNi18 | RPRWSHRSRR | >128 | >128 | >128 | non-toxic |
| RaOm1 | GLLSGILGAGKK | >128 | >128 | >128 | non-toxic |
| RaSi2 | FPFPFGRR | >128 | >128 | >128 | non-toxic |
| AnFl1 | DNKWQNVHFHRSAVTGPTSFSFSHK | >128 | >128 | >128 | non-toxic |
| ApMe1 | VKCRVRR | >128 | >128 | >128 | non-toxic |
| ApMe2 | GAHKEVFKRDTALTKEAAKKAKK | >128 | >128 | >128 | non-toxic |
| ApMe3 | GWGLINIKIPPVLHKVSVPLVSKR | >128 | >128 | >128 | non-toxic |
| ApMe4 | KHHHIKLRHERHRRYILKSLI | >128 | >128 | >128 | non-toxic |
| ApMe5 | SILSTLSHKR | >128 | >128 | >128 | non-toxic |
| ApMe6 | RARKIRRRRGSLRHCVTIPSTPSGR | >128 | >128 | >128 | non-toxic |
| BoAr1 | AAGAGKVTKSAQKAQKAK | >128 | >128 | >128 | non-toxic |
| BoAr2 | ATAAECLKHPWLKIKK | >128 | >128 | >128 | non-toxic |
| BoAr3 | IIRATAAECLKHPWLKIKK | >128 | >128 | >128 | non-toxic |
| BoAr4 | SVASLAKNSAWPVSLKR | >128 | >128 | >128 | non-toxic |
| BoAr5 | VTISIARRVSSHKRG | >128 | >128 | >128 | non-toxic |
| BoCo1 | NKIKFINKYVKKVQLKKILVKS | >128 | >128 | >128 | non-toxic |
| CaCa3 | KHHHIKLRHGRHRRSVLRTLV | >128 | >128 | >128 | non-toxic |
| MiDe1 | VMLPKFKR | >128 | >128 | >128 | non-toxic |
| NaVi1 | TPLSDIFRGQLRSRVSR | >128 | >128 | >128 | non-toxic |
| NaVi2 | SSLSPLSSSSGLGKKKKRKSKRASR | >128 | >128 | >128 | non-toxic |
| NaVi4 | GSSSRSCRCIRLSRLSSKRT | >128 | >128 | >128 | non-toxic |
| PoSn1 | ISIKEALEHSFFHTVPRKWCKKH | >128 | >128 | >128 | non-toxic |
| PaVa1Q5K | RPRPKQVPPRPPHPRLRR | >128 | >128 | >128 | non-toxic |
| PaVa1Q6R | RPRPQRVPPRPPHPRLRR | >128 | >128 | >128 | non-toxic |
| PaVa1P2R | RRRPQQVPPRPPHPRLRR | >128 | >128 | >128 | non-toxic |
| PaVa1V7R | RPRPQQRPPRPPHPRLRR | >128 | >128 | >128 | non-toxic |
| PaVa1H13R | RPRPQQVPPRPPRPRLRR | >128 | >128 | >128 | non-toxic |
| PaVa1P4K | RPRKQQVPPRPPHPRLRR | >128 | >128 | >128 | non-toxic |
| PaVa1H13K | RPRPQQVPPRPPKPRLRR | >128 | >128 | >128 | non-toxic |
| PeNi4G4K | GLLKKVLGVGKKVLC | >128 | >128 | >128 | non-toxic |
| PeNi4V9R | GLLGKVLGRGKKVLC | >128 | >128 | >128 | non-toxic |
| PeNi4G10R | GLLGKVLGVRKKVLC | >128 | >128 | >128 | non-toxic |
| PeNi4V13K | GLLGKVLGVGKKKLC | >128 | >128 | >128 | non-toxic |
| CaCa2W22K | GLLGKVRGVGKKVLC | >128 | >128 | >128 | non-toxic |
| TeBi1P5K | KIKIKWGKVKDFLVGGMKAVGKK | >128 | >128 | >128 | non-toxic |
| TeBi1V14K | KIKIPWGKVKDFLKGGMKAVGKK | >128 | >128 | >128 | non-toxic |
| TeBi1G21K | KIKIPWGKVKDFLVGGMKAVKKK | >128 | >128 | >128 | non-toxic |
| TeBi1I4K | KIKKPWGKVKDFLVGGMKAVGKK | >128 | >128 | >128 | non-toxic |
| TeBi1A19K | KIKIPWGKVKDFLVGGMKKVGKK | >128 | >128 | >128 | non-toxic |
| TeBi1D11K | KIKIPWGKVKKFLVGGMKAVGKK | >128 | >128 | >128 | non-toxic |
| TeBi1W6K | KIKIPKGKVKDFLVGGMKAVGKK | >128 | >128 | >128 | non-toxic |
| TeBi1V9K | KIKIPWGKKKDFLVGGMKAVGKK | >128 | >128 | >128 | non-toxic |
| TeRu4S6K | SWLSKKVKKLVNKKNYTRLEKLAKKKLFNE | >128 | >128 | >128 | non-toxic |
| TeRu4F28K | SWLSKSVKKLVNKKNYTRLEKLAKKKLKNE | >128 | >128 | >128 | non-toxic |
| TeRu4N29K | SWLSKSVKKLVNKKNYTRLEKLAKKKLFKE | >128 | >128 | >128 | non-toxic |
| TeRu4S1R | RWLSKSVKKLVNKKNYTRLEKLAKKKLFNE | >128 | >128 | >128 | non-toxic |
| TeRu4N15K | SWLSKSVKKLVNKKKYTRLEKLAKKKLFNE | >128 | >128 | >128 | non-toxic |
| AmMa1T5K | GILDKLKQLGKAAVQGLLSKAACKLAKTC | >128 | >128 | >128 | non-toxic |
| AmMa1Q8K | GILDTLKKLGKAAVQGLLSKAACKLAKTC | >128 | >128 | >128 | non-toxic |
| AmMa1A12K | GILDTLKQLGKKAVQGLLSKAACKLAKTC | >128 | >128 | >128 | non-toxic |
| AmMa1A21K | GILDTLKQLGKAAVQGLLSKKACKLAKTC | >128 | >128 | >128 | non-toxic |
| ApCe1L22R | GIYTGRLLPVYIPQPRPPHPRRRR | >128 | >128 | >128 | non-toxic |
| ApCe1Y3R | GIRTGRLLPVYIPQPRPPHPRLRR | >128 | >128 | >128 | non-toxic |
| ApCe1G5R | GIYTRRLLPVYIPQPRPPHPRLRR | >128 | >128 | >128 | non-toxic |
| ApCe1V10R | GIYTGRLLPRYIPQPRPPHPRLRR | >128 | >128 | >128 | non-toxic |
| ApCe1P9R | GIYTGRLLRVYIPQPRPPHPRLRR | >128 | >128 | >128 | non-toxic |
| CaCa2D21K | FIKTQVLKHLVAGVRVARGLKWKWR | >128 | >128 | >128 | non-toxic |
| CaCa2T4K | FIKKQVLKHLVAGVRVARGLDWKWR | >128 | >128 | >128 | non-toxic |
| CaCa2W22K | FIKTQVLKHLVAGVRVARGLDKKWR | >128 | >128 | >128 | non-toxic |
| CaCa2H9K | FIKTQVLKKLVAGVRVARGLDWKWR | >128 | >128 | >128 | non-toxic |
| CaCa2A12K | FIKTQVLKHLVKGVRVARGLDWKWR | >128 | >128 | >128 | non-toxic |
| CaCa4A6R | RRFFFRTAPCGYSRKFCKITRRKR | >128 | >128 | >128 | non-toxic |
| CaCa4T7R | RRFFFARAPCGYSRKFCKITRRKR | >128 | >128 | >128 | non-toxic |
| CaCa4F5K | RRFFKATAPCGYSRKFCKITRRKR | >128 | >128 | >128 | non-toxic |
| CaCa4C10R | RRFFFATAPRGYSRKFCKITRRKR | >128 | >128 | >128 | non-toxic |
| MyGu1A6K | RRAIFKSIRGYLGLRKR | >128 | >128 | >128 | non-toxic |
| MyGu1Y11K | RRAIFASIRGKLGLRKR | >128 | >128 | >128 | non-toxic |
| MyGu1L14K | RRAIFASIRGYLGKRKR | >128 | >128 | >128 | non-toxic |
| MyGu1G13K | RRAIFASIRGYLKLRKR | >128 | >128 | >128 | non-toxic |
| MyGu1A3K | RRKIFASIRGYLGLRKR | >128 | >128 | >128 | non-toxic |
| NaVi3L4K | KLFKTLWKLKR | >128 | >128 | >128 | non-toxic |
| NaVi3T5R | KLFLRLWKLKR | >128 | >128 | >128 | non-toxic |
| NaVi3W7K | KLFLTLKKLKR | >128 | >128 | >128 | non-toxic |
| NaVi3L6R | KLFLTRWKLKR | >128 | >128 | >128 | non-toxic |
| OdMa2G19K | GLLRGILGAGKKIVCGLSKLC | >128 | >128 | >128 | non-toxic |
| OdMa2G16K | GLLRGILGAGKKIVCKLSGLC | >128 | >128 | >128 | non-toxic |
| OdMa2L3R | GLRRGILGAGKKIVCGLSGLC | >128 | >128 | >128 | non-toxic |
| OdMa2G5R | GLLRRILGAGKKIVCGLSGLC | 128 | 128 | 32-64 | toxic |
| OdMa2A9R | GLLRGILGRGKKIVCGLSGLC | >128 | >128 | >128 | non-toxic |
| OdMa2C15K | GLLRGILGAGKKIVKGLSGLC | >128 | >128 | >128 | non-toxic |
| OdMa2S18K | GLLRGILGAGKKIVCGLKGLC | >128 | >128 | >128 | non-toxic |
| OdMa12T5K | GFMDKAKNVAKNVAVTLLYNLKCKITKAC | >128 | >128 | >128 | non-toxic |
| OdMa12N20K | GFMDTAKNVAKNVAVTLLYKLKCKITKAC | >128 | >128 | >128 | non-toxic |
| LiVe2I5K | GWLDKAKKVASVVAGLGKR | >128 | >128 | >128 | non-toxic |
| LiVe2S11K | GWLDIAKKVAKVVAGLGKR | >128 | >128 | >128 | non-toxic |
| LiVe2A14K | GWLDIAKKVASVVKGLGKR | >128 | >128 | >128 | non-toxic |
| DeNo1001 | DLLSGLGKAAKKVAKTVLKNLLKC | >128 | >128 | >128 | non-toxic |
| DeNo1002 | NLLDTLKNLAKKLAKKLLKKLLKKL | 64-128 | 128 | 128 | toxic |
| DeNo1003 | NLLSTLLDAAKKAAKGAAKSAAKKLAKKLAKKL | >128 | >128 | >128 | non-toxic |
| DeNo1004 | HLLSGLLSAAKKAAKKAAKKALKKLLKKLLKKL | >128 | >128 | >128 | non-toxic |
| DeNo1005 | GLFSLLKKLLKKLLKKLLKKLLKKLLKKL | >128 | >128 | >128 | non-toxic |
| DeNo1006 | NLLDTLKKKAKKVAKKVLKKLLKKLLKKL | >128 | >128 | >128 | non-toxic |
| DeNo1007 | FLPSIIKGAAKKLPKIFCKILKKC | 128->128 | >128 | >128 | non-toxic |
| DeNo1008 | GLLSLLKKLLKKLLKKLLKKL | 8.0-16.0 | 32 | 16 | toxic |
| DeNo1009 | DLLKTLGKAAKKAAKTALKAALKGLLKKLAKKL | >128 | >128 | >128 | non-toxic |
| DeNo1010 | VLGGLLKKLLKKLLKKL | 64 | 128 | 128 | toxic |
| DeNo1011 | HLLSLLKKAAKKLLKKLLKKLAKKL | 32 | 32 | 32-64 | toxic |
| DeNo1012 | CLLDTLKCVAKGVAGTLLDTLKCKITGKC | >128 | >128 | >128 | non-toxic |
| DeNo1013 | KIFGKILKKLLKKLLKKLLKKL | 64-128 | 64 | 64 | toxic |
| DeNo1014 | ALPSLLKKLAKKLAKKLLKKLLKKLLKKLLKKL | >128 | 128->128 | 128->128 | toxic |
| DeNo1015 | NLLDTLKNVAKNVAKNVLDTLKCKITCKC | >128 | >128 | >128 | non-toxic |
| DeNo1016 | FLPIIAGLAAKFLPKIFCKITKKC | 4 | 8-16 | 8 | toxic |
| DeNo1017 | FLPIIAGLAAKLLPKLFCKITKKC | 8-16 | 16 | 16 | toxic |
| DeNo1018 | WLPKIAGKIAGKLLKKLLKKIKKK | 128 | 128 | 128 | toxic |
| DeNo1019 | FLPKIAGKAAKKLPKIFCKITKKC | >128 | >128 | >128 | non-toxic |
| DeNo1020 | TLPDVAKNVAKNVAKTVLDTLKCKITGKC | >128 | >128 | >128 | non-toxic |
| DeNo1021 | KLFGKLLKKLKKILKKIAKKIKKKL | >128 | >128 | >128 | non-toxic |
| DeNo1022 | GLLSLLKKIGKKIGKLL | 128 | >128 | >128 | non-toxic |
| DeNo1023 | DLLKTLKKIAKKLLKTLLKKLLKKLLKKL | >128 | >128 | >128 | non-toxic |
| DeNo1024 | KLFGKILGKIAKKILGKILGALLSKLLSAL | >128 | >128 | >128 | non-toxic |
| DeNo1025 | DLLSCLKKKGKCVLKNL | >128 | >128 | >128 | non-toxic |
| DeNo1026 | RLPSLFKKLFKKIAKVVGKIAKKILKK | 128 | 128->128 | 128->128 | toxic |
| DeNo1027 | RLPSIIPGIAGKLGGLLGGLLKGL | >128 | >128 | >128 | non-toxic |
| DeNo1028 | CLPSLLPSLFKKL | >128 | >128 | >128 | non-toxic |
| DeNo1029 | SLPSILSGIAGKL | >128 | >128 | >128 | non-toxic |
| DeNo1030 | RLPRIFRGIRGKL | >128 | >128 | >128 | non-toxic |
| DeNo1031 | PLPPIIPGIAGKLLGGLLGLLKKL | 128 | 128 | 128 | toxic |
| DeNo1032 | YLPSVLPSVLKPL | >128 | >128 | >128 | non-toxic |
| DeNo1033 | PLPPIIPGLASGLLSGLC | >128 | >128 | >128 | non-toxic |
| DeNo1034 | KLPSIIKAAAKALPKLF | >128 | >128 | >128 | non-toxic |
| DeNo1035 | QLPRIAGKIAKKL | >128 | >128 | >128 | non-toxic |
| DeNo1036 | QLPSVLPAIAKAL | >128 | >128 | >128 | non-toxic |
| DeNo1037 | CLPSILC | >128 | >128 | >128 | non-toxic |
| DeNo1038 | MLPSIAGAAAKGLPKLFCKITKKC | >128 | >128 | >128 | non-toxic |
| DeNo1039 | MLPKIFGKIFKKILKKILKKILKKILKKLLKKL | 32 | 64 | 32 | toxic |
| DeNo1040 | MLPSILGALLKLL | >128 | >128 | >128 | non-toxic |
| DeNo1041 | MLPKIAGKIAKKL | >128 | >128 | >128 | non-toxic |
| DeNo1042 | MLPKIAGAIAKLL | >128 | >128 | >128 | non-toxic |
| DeNo1043 | WLPKIAGKIAGKL | >128 | >128 | >128 | non-toxic |
| DeNo1044 | CLPSILCKITKKC | >128 | >128 | >128 | non-toxic |
| DeNo1045 | FLPKIFKKIAKKL | >128 | >128 | >128 | non-toxic |
| DeNo1046 | VLGSLLKGLLKKL | >128 | >128 | >128 | non-toxic |
| DeNo1047 | ALPSIIKGLLKKL | >128 | >128 | >128 | non-toxic |
| DeNo1048 | LLPSLLKGLLKKL | >128 | >128 | >128 | non-toxic |
| DeNo1049 | ALLSLLKKLLKKL | >128 | >128 | >128 | non-toxic |
| DeNo1050 | FLPKIAGKIAGKL | >128 | >128 | >128 | non-toxic |
| DeNo1051 | ALPSLLKKLLKKL | >128 | >128 | >128 | non-toxic |
| DeNo1052 | YLPSVLKGLLKKL | >128 | >128 | >128 | non-toxic |
| DeNo1053 | LLPSLLKGLAKKL | >128 | >128 | >128 | non-toxic |
| DeNo1054 | QLPKIAGKIAKKL | >128 | >128 | >128 | non-toxic |
| DeNo1055 | FLPKIFKKIAKKI | >128 | >128 | >128 | non-toxic |
| DeNo1056 | GLLSLLKKLLKKL | >128 | >128 | >128 | non-toxic |
| DeNo1057 | ILGKLLKKLLKKL | >128 | >128 | >128 | non-toxic |
| DeNo1058 | FLPKIAGKIAKKL | >128 | >128 | >128 | non-toxic |
| RaCa2 | FFPIIARLAAKVIPSLVCAVTKKC | 8 | 32 | 16 | toxic |
| RaCa2I4K | FFPKIARLAAKVIPSLVCAVTKKC | 128 | 128 | 128 | toxic |
| RaCa2P14K | FFPIIARLAAKVIKSLVCAVTKKC | 8 | 4-8 | 16 | toxic |
| RaCa2S15K | FFPIIARLAAKVIPKLVCAVTKKC | 16 | 16 | 16 | toxic |
| RaCa2A19K | FFPIIARLAAKVIPSLVCKVTKKC | 32 | 32 | 32-64 | toxic |
| RaCa2A19KI4K | FFPKIARLAAKVIPSLVCKVTKKC | 128 | 128 | 128 | toxic |
| RaCa2S15KA19KT21K | FFPIIARLAAKVIPKLVCKVKKKC | 128 | 128 | 128 | toxic |
| RaCa2I4KT21KA19K | FFPKIARLAAKVIPSLVCKVKKKC | 128 | 128 | 128 | toxic |
| RaCa2A19KI4KP3R | FFRKIARLAAKVIPSLVCKVTKKC | 32 | 32 | 64 | toxic |
| RaCa2C24S | FFPIIARLAAKVIPSLVCAVTKKS | 32 | 16 | 32 | toxic |
| RaCa2C18SC24S | FFPIIARLAAKVIPSLVSAVTKKS | 128 | 128 | 128 | toxic |
| RaCa2t1_18 | FFPIIARLAAKVIPSLVC | 64 | 64 | 32-64 | toxic |
| RaCa2P3RS15Kt1_18 | FFRIIARLAAKVIPKLVC | 16 | 8 | 16-32 | toxic |
| RaCa2I4KS15Kt1_18 | FFPKIARLAAKVIPKLVC | 32 | 16 | 64 | toxic |
| RaCa2P3RI4KS15Kt1_18 | FFRKIARLAAKVIPKLVC | 16 | 8 | 16 | toxic |
| RaCa2C18St1_18 | FFPIIARLAAKVIPSLVS | ≥128 | >128 | >128 | non-toxic |
| RaCa2P3RI4KS15C18SKt1_18 | FFRKIARLAAKVIPKLVS | 128 | 128 | >128 | toxic |

**References**

Chung, J., Gulcehre, C., Cho, K., & Bengio, Y. (2014, December 11). *Empirical Evaluation of Gated Recurrent Neural Networks on Sequence Modeling*. arXiv. Retrieved from http://arxiv.org/abs/1412.3555
