# Supplementary material for: Structure‐aware deep learning model for peptide toxicity prediction
Source: Protein Sci. 2024 Jun 22;33(7):e5076. doi: 10.1002/pro.5076 (PMC11193153; doi:10.1002/pro.5076)
Supplement: Supplementary file 2 — Data S2. Supporting information. [file PRO-33-e5076-s001.docx]

**Supplementary Materials**

**Structure-aware deep learning model for peptide toxicity prediction**

Hossein Ebrahimikondori^1,2^, Darcy Sutherland^1,3,4^, Anat Yanai^1,3^, Amelia Richter^1,3^, Ali Salehi^1,3^, Chenkai Li^1,2^, Lauren Coombe^1^, Monica Kotkoff^1^, René L. Warren^1^, and Inanc Birol^1,3,4,5, *^

^1^ Canada’s Michael Smith Genome Sciences Centre, BC Cancer Agency, Vancouver, BC, V5Z 4S6, Canada

^2^ Bioinformatics Graduate Program, University of British Columbia, Vancouver, BC, V6T 1Z4, Canada

^3^ Public Health Laboratory, British Columbia Centre for Disease Control, Vancouver, BC, V5Z 4R4, Canada

^4^ Department of Pathology and Laboratory Medicine, University of British Columbia, Vancouver, BC, V6T 1Z4, Canada

^5^ Department of Medical Genetics, University of British Columbia, Vancouver, BC, V6H 3N1, Canada

* Correspondence: Inanc Birol (ibirol@bcgsc.ca)


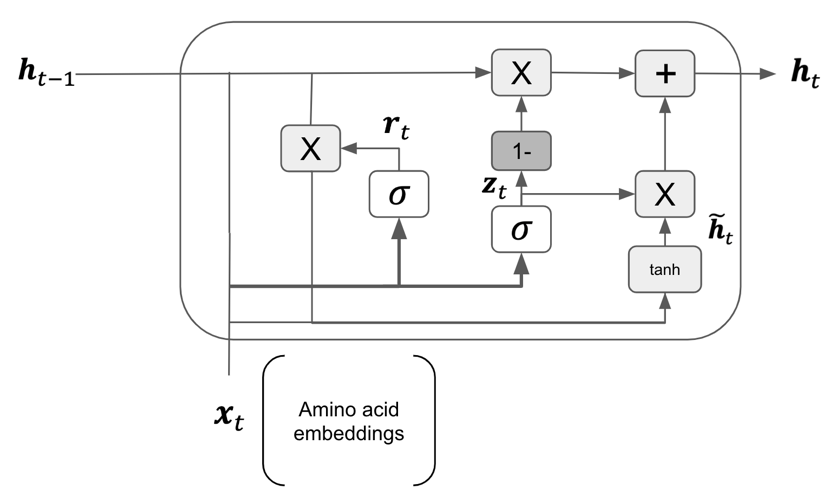


**Supplementary Figure 1**. schematic diagram of GRUs (Chung, Gulcehre, Cho, & Bengio, 2014).

For the $t$-th residue in a sequence, the $j$-th unit in the hidden vector $\boldsymbol{h}_{t}^{(j)}$ is computed as a linear combination of the $j$-th previous hidden vector in the sequence $\boldsymbol{h}_{t-1}^{(j)}$and the candidate vector ${\tilde{\boldsymbol{h}}}_{t}^{(j)}$:

$$\boldsymbol{h}_{t}^{(j)}=\left( 1-\boldsymbol{z}_{t}^{\left( j \right)} \right)\boldsymbol{h}_{t-1}^{\left( j \right)}+ \boldsymbol{z}_{t}^{\left( j \right)}{\tilde{\boldsymbol{h}}}_{t}^{(j)}$$

as described in (Chung et al., 2014). The update gate $\boldsymbol{z}_{t}^{\left( j \right)}$ decides how much the content $\boldsymbol{h}_{t}^{(j)}$ should be updated. The update gate is computed as follows,

$$\boldsymbol{z}_{t}^{\left( j \right)}=\sigma\left( \boldsymbol{W}_{z}\boldsymbol{x}_{t}+\boldsymbol{U}_{z}\boldsymbol{h}_{t-1} \right)^{\left( j \right)},$$

Where $\sigma$ indicates sigmoid function, $\boldsymbol{W}_{z}$ and $\boldsymbol{U}_{z}$ are learnable weight matrices, and $\boldsymbol{x}_{t}$ is the given $t$-th residue’s input embeddings. The candidate vector ${\tilde{\boldsymbol{h}}}_{t}^{(j)}$ is computed based on the input and how much should we forget from the previously computed hidden state.

$${\tilde{\boldsymbol{h}}}_{t}^{(j)}=\tanh\left( \boldsymbol{W}\boldsymbol{x}_{t}+\boldsymbol{U}(\boldsymbol{r}_{t}\odot\boldsymbol{h}_{t-1}) \right)^{\left( j \right)}$$

where $\boldsymbol{r}_{t}$ is a vector of reset gates for each unit and $\odot$ indicates element-wise multiplication. Each reset gate $\boldsymbol{r}_{t}^{\left( j \right)}$ is computed similarly to upadate gate as follows:

$$\boldsymbol{r}_{t}^{\left( j \right)}=\sigma\left( \boldsymbol{W}_{r}\boldsymbol{x}_{t}+\boldsymbol{U}_{r}\boldsymbol{h}_{t-1} \right)^{\left( j \right)}$$

**Supplementary equations 1**. **Gaussian radial basis functions**

$$\mu_{i}=d_{min}+i\times\frac{d_{max}-d_{min}}{count}$$

$$\sigma=\frac{d_{max}-d_{min}}{count}$$

$${rbf}_{i}\left( d \right)=\mathrm{ex}p \left\{ -\left( \frac{d-\mu_{i}}{\sigma} \right)^{2} \right\}$$

We used 16 Gaussian radial basis functions ($count=16$) with $d_{min}=0.0$ and $d_{max}=20$ Angstroms.

**Supplementary equations 2**. **sinusoidal encoding**

$$PE\left( distance, 2i \right)=\sin\left( \frac{distance}{{10000}^{\frac{2i}{d}}} \right)$$

$$PE\left( distance, 2i+1 \right)=\cos\left( \frac{distance}{{10000}^{\frac{2i}{d}}} \right)$$

Where $d$ ($=16$) is the number of embeddings and $i\in[0,16)$ indicates the $i$-th dimension.


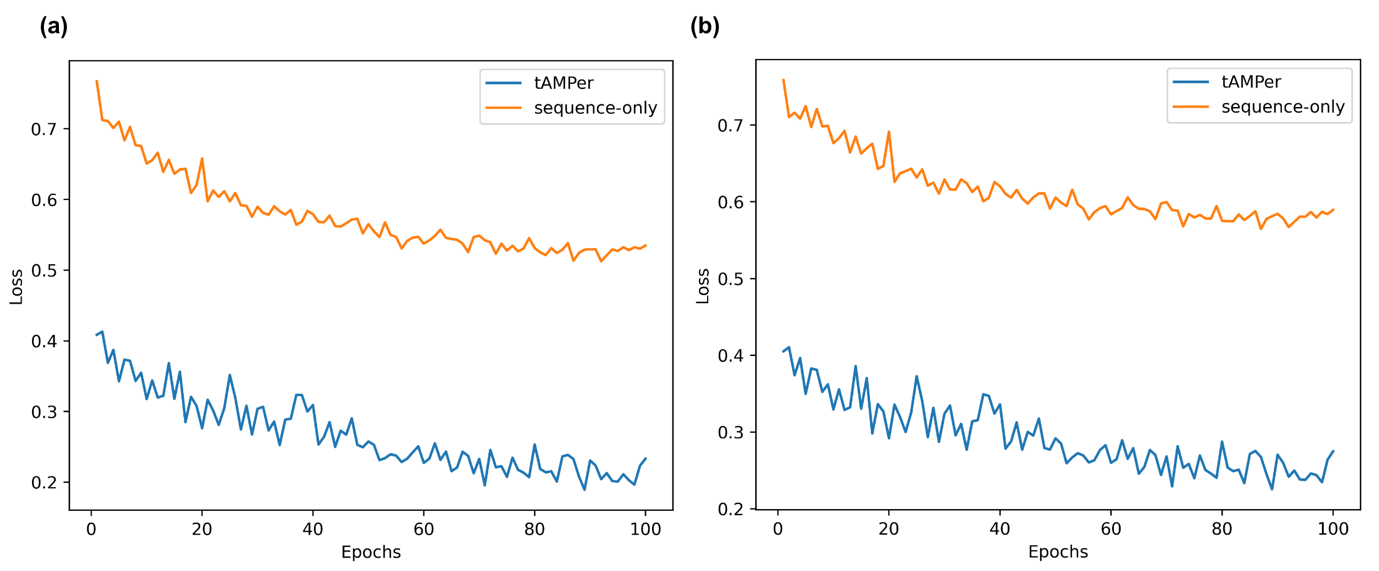


**Supplementary Figure 2**. **(a) Training and (b) validation loss curves for secondary structure prediction using tAMPer and sequence-only variant.** The orange curves represent the model's performance when utilizing only sequential features, while the blue curves depict the model's performance when employing both sequential and structural features. The training error for the sequence-only model remains relatively high even after 100 epochs, suggesting its difficulty in capturing relevant features for the given task, as anticipated. However, the results indicate that the structural features are highly effective in accurately predicting the secondary structure for each residue in the peptide sequences. Adding $\mathcal{L}_{ss}$ in tAMPer's loss function compels the model to include the extracted structural features as a component of the abstract representation of amino acids.

**Supplementary Table 1**. **Performance comparison of tAMPer based on different values of** $\boldsymbol{\lambda}$ **and** $\boldsymbol{d}_{\boldsymbol{max}}$ **on the validation dataset.** The metrics are presented as percentages. Highest value for each metric is bolded.

| $d_{max}$ | $\lambda$ | **Sensitivity** | **Specificity** | **F1** | **MCC** | **auROC** | **auPRC** |
| --- | --- | --- | --- | --- | --- | --- | --- |
| 8 | 0.0 | 68.9 | 64.7 | 55.8 | 31.1 | 71.4 | 50.3 |
|  | 0.1 | 70.8 | 68.6 | 58.9 | 36.8 | 74.0 | 54.9 |
|  | 0.2 | 61.2 | 75.8 | 57.0 | 35.7 | 73.9 | 57.2 |
|  | 0.3 | 65.9 | 71.3 | 57.5 | 35.1 | 73.8 | 54.4 |
|  | 0.4 | 65.9 | 70.1 | 56.8 | 33.8 | 72.4 | 52.8 |
|  | 0.5 | 65.6 | 68.9 | 55.9 | 32.3 | 72.2 | 52.4 |
| 10 | 0.0 | 67.5 | 68.2 | 56.8 | 33.4 | 72.7 | 52.6 |
|  | 0.1 | 63.7 | 72.7 | 56.8 | 34.6 | 73.0 | 52.7 |
|  | 0.2 | 63.7 | 75.9 | 58.7 | 38.1 | **75.1** | 56.4 |
|  | 0.3 | 54.1 | 76.9 | 52.7 | 30.6 | 71.7 | 50.5 |
|  | 0.4 | 60.1 | 75.6 | 56.1 | 34.5 | 73.6 | **57.3** |
|  | 0.5 | 60.1 | 73.6 | 55.0 | 32.3 | 72.7 | 51.9 |
| 12 | 0.0 | 59.6 | 74.3 | 55.1 | 32.6 | 72.4 | 54.0 |
|  | 0.1 | 63.9 | 73.4 | 57.4 | 35.6 | 74.0 | 53.0 |
|  | 0.2 | 67.5 | 73.3 | **59.6** | **38.7** | 74.9 | 55.7 |
|  | 0.3 | 58.2 | 73.4 | 53.7 | 30.4 | 71.1 | 49.2 |
|  | 0.4 | 56.8 | 74.9 | 53.5 | 30.8 | 72.1 | 52.5 |
|  | 0.5 | 68.9 | 65.1 | 56.0 | 31.6 | 72.2 | 50.6 |
| 20 | 0.0 | 52.7 | **82.8** | 55.3 | 36.6 | 72.6 | 53.2 |
|  | 0.1 | 66.7 | 70.1 | 57.3 | 34.6 | 73.0 | 55.5 |
|  | 0.2 | **71.0** | 65.3 | 57.3 | 33.7 | 72.7 | 51.4 |
|  | 0.3 | 63.1 | 73.2 | 56.8 | 34.6 | 74.0 | 53.6 |
|  | 0.4 | 66.4 | 70.0 | 57.0 | 34.2 | 73.8 | 53.1 |
|  | 0.5 | 54.4 | 81.7 | 55.8 | 36.6 | 74.8 | 55.0 |

**Supplementary Table 2. Peptide hemolysis dataset.** Each hemolysis assessment was performed in triplicate (N=3) to determine the concentration required for 50% hemolysis of red blood cells (HC50). A peptide is labeled toxic if it exhibits an HC50 value of less than or equal to 128 µg/ml in at least two technical replicates.

| **Peptide name** | **Sequence** | **HC50 (µg/ml)** | | | **Label** |
| --- | --- | --- | --- | --- | --- |
|  |  | **N=1** | **N=2** | **N=3** |  |
| OdMa12 | GFMDTAKNVAKNVAVTLLYNLKCKITKAC | >128 | >128 | >128 | non-toxic |
| PeNi7 | VIPFVASVAAEMMHHVYCAASKRCKN | >128 | >128 | >128 | non-toxic |
| PeNi10 | GLLLDTVKGAAKNVAGILLNKLKCKVTGDC | >128 | >128 | >128 | non-toxic |
| PeNi11 | GILTDTLKGAAKNVAGVLLDKLKCKITGGC | >128 | >128 | >128 | non-toxic |
| PeNi14 | GLWTTIKEGVKNFSVGVLDKIRCKITGGC | >128 | >128 | >128 | non-toxic |
| PeNi16 | ATAWKVPPGLQPIRPIRIRPLCGNDKS | >128 | >128 | >128 | non-toxic |
| RaOm5 | AGYSRMIRRPPGFSPFRVAPASSLKR | >128 | >128 | >128 | non-toxic |
| RaSy2 | EEQRFLPVVAGLAAKVLPSIICAVTKKC | >128 | >128 | >128 | non-toxic |
| BoAr6 | GILRLVTRRFRFSPTNLNRYTVARLVSGVP | >128 | >128 | >128 | non-toxic |
| TeRu3 | AVLSFVHKLFLNFLHVDTSKGKCRATLQ | >128 | >128 | >128 | non-toxic |
| TeRu4 | SWLSKSVKKLVNKKNYTRLEKLAKKKLFNE | >128 | >128 | >128 | non-toxic |
| PaVa2 | KYHHIKLRHGRHRRTIH | >128 | >128 | >128 | non-toxic |
| PaVa3 | ITEPVGTKAPTFTSELRGGWLKKR | >128 | >128 | >128 | non-toxic |
| PaVi1 | WALRWKTR | >128 | >128 | >128 | non-toxic |
| PoRo1 | VAAFAIIGCLCCRRPRR | >128 | >128 | >128 | non-toxic |
| PoSn2 | TALKSLSILKKLAKLNM | >128 | >128 | >128 | non-toxic |
| TeRu1 | VPFGLKPR | >128 | >128 | >128 | non-toxic |
| TeRu2 | AFVRILCYCCPRRIKRR | >128 | >128 | >128 | non-toxic |
| VeSi1 | FILHAKKTRSAK | >128 | >128 | >128 | non-toxic |
| OdMa13 | GFMDTAKNVAKNVAVTLLDNLKCKITKAC | >128 | >128 | >128 | non-toxic |
| OdTo4 | KLMIPRKKRGIFGGLLKVGKKIACGLSGLC | >128 | >128 | >128 | non-toxic |
| PeNi8 | GILLNTLKGAAKNVAGVLLDKLKCKITGGC | >128 | >128 | >128 | non-toxic |
| LiVe1 | GWLDIAKKVASVVAGIVKR | >128 | >128 | >128 | non-toxic |
| LiVe2 | GWLDIAKKVASVVAGLGKR | >128 | >128 | >128 | non-toxic |
| OdMa1 | GLLSGILGAGKKIVCGFSGLC | >128 | >128 | >128 | non-toxic |
| OdMa3 | GLLSGLLGAGKKIVCGLSGMC | >128 | >128 | >128 | non-toxic |
| OdMa4 | GILSGLLGAGKKIVC | >128 | >128 | >128 | non-toxic |
| OdMa6 | GLLSGVLGVGKKIVCGLSGLC | >128 | >128 | >128 | non-toxic |
| OdMa9 | GLISGILGAGKKVLC | >128 | >128 | >128 | non-toxic |
| OdMa10 | GLISGILGAGKKVLCGLSGLC | >128 | >128 | >128 | non-toxic |
| OdTo1 | GILSGLLGAGKKLACGLIGLC | ≥128 | >128 | >128 | non-toxic |
| OdTo2 | GIFGGHLKVGKKIACGLSGLC | >128 | >128 | >128 | non-toxic |
| OdTo3 | GIFGGLLKEGKKIACGLSGLC | >128 | >128 | >128 | non-toxic |
| PeNi2 | GLLGKVLGVGKKVLCVVSGLC | >128 | >128 | >128 | non-toxic |
| PeNi3 | GIFSLIKGAAKVVAKGLG | >128 | >128 | >128 | non-toxic |
| PeNi4 | GLLGKVLGVGKKVLC | >128 | >128 | >128 | non-toxic |
| PeNi5 | GLLGKVLGVGKKVLCGVTGRERCQ | >128 | >128 | >128 | non-toxic |
| RaOm2 | GILSGLLGAGKKIVCGLSGMC | >128 | >128 | >128 | non-toxic |
| RaOm3 | GIFSLIKGAAKVVAKGLGK | >128 | >128 | >128 | non-toxic |
| RaOm4 | GLLGKVLGVGKKVLCGVSGRC | >128 | >128 | >128 | non-toxic |
| RaSi1 | GLVGKLVKGGLKLIGHVANG | >128 | >128 | >128 | non-toxic |
| AnFl2 | GILRSLGWIQMPRSRRRHR | >128 | >128 | >128 | non-toxic |
| ApCe1 | GIYTGRLLPVYIPQPRPPHPRLRR | >128 | >128 | >128 | non-toxic |
| BoUs1 | RKIIAVSVHKLCRVKR | >128 | >128 | >128 | non-toxic |
| CaCa1 | FACPIGFFRLKR | >128 | >128 | >128 | non-toxic |
| CaCa2 | FIKTQVLKHLVAGVRVARGLDWKWR | >128 | >128 | >128 | non-toxic |
| CaCa4 | RRFFFATAPCGYSRKFCKITRRKR | >128 | >128 | >128 | non-toxic |
| DiLo1 | GAFVLWGPTPRPRRR | >128 | >128 | >128 | non-toxic |
| MyGu1 | RRAIFASIRGYLGLRKR | >128 | >128 | >128 | non-toxic |
| NaVi3 | KLFLTLWKLKR | >128 | >128 | >128 | non-toxic |
| PaVa1 | RPRPQQVPPRPPHPRLRR | >128 | >128 | >128 | non-toxic |
| CLIB_denovo9 | TLPDVAKGAAKGLAKTALDVLKCKLKGVC | >128 | >128 | >128 | non-toxic |
| CLIB_denovo10 | VLGSLLKGLGKLLGKILGKIGKKIGKCVGKC | 128 | 128 | >128 | toxic |
| CLIB_denovo19 | GLLSLLKKLLKKLC | >128 | 128 | 64 | toxic |
| CLIB_denovo14 | GMLSKIKGALKKVAKKILKKL | >128 | >128 | >128 | non-toxic |
| CLIB_denovo16 | GTLSKLLKKLFKKILKKL | ≥128 | 128 | 128 | toxic |
| CLIB_denovo11 | GVLSTLKKKLKKLLKKLLKTLLKKI | 128 | 64 | 32-64 | toxic |
| CLIB_denovo12 | RFGSIIKNVGKNVLKTLLCKIKKKC | >128 | >128 | 128 | non-toxic |
| CLIB_denovo13 | GWLSKLKKKGKKIGKAIKKIGKKF | >128 | >128 | >128 | non-toxic |
| CLIB_deno38 | TLPSLLSGLLKKL | >128 | >128 | >128 | non-toxic |
| CLIB_denovo26 | GDLSKLKGKGKKIGGKLLGGLKKKIKGVC | >128 | >128 | >128 | non-toxic |
| CLIB_denovo27 | LVGKLLKKLGKKIKKIF | >128 | >128 | >128 | non-toxic |
| CLIB_denovo17 | GWLSKLKKKGKKIGKAIKKIGKKFC | >128 | >128 | >128 | non-toxic |
| CLIB_denovo29 | WDWLKKKGKGVAGLVAKALKAALKAL | 128 | 128 | 128 | toxic |
| CLIB_denovo18 | GMLSKIKGALKKVAKKILKKLC | ≥128 | ≥128 | >128 | toxic |
| CLIB_denovo20 | GTLSKLLKKLFKKILKKLC | 64 | 128 | 128 | toxic |
| CLIB_denovo30 | DVLDTLKNAAGKLAKKLLKKLLKKI | >128 | >128 | >128 | non-toxic |
| RaCa3 | GLWETIKTTGKSIALNLLDKIKCKIAGGCPP | >128 | >128 | 128 | non-toxic |
| RaCa3T5K | GLWEKIKTTGKSIALNLLDKIKCKIAGGCPP | >128 | >128 | >128 | non-toxic |
| RaCa3T8K | GLWETIKKTGKSIALNLLDKIKCKIAGGCPP | 128 | 128 | ≥128 | toxic |
| RaCa3S12K | GLWETIKTTGKKIALNLLDKIKCKIAGGCPP | >128 | >128 | 64-128 | non-toxic |
| RaCa3A26R | GLWETIKTTGKSIALNLLDKIKCKIRGGCPP | >128 | >128 | >128 | non-toxic |
| RaCa3G28K | GLWETIKTTGKSIALNLLDKIKCKIAGKCPP | >128 | >128 | >128 | non-toxic |
| RaCa3P31K | GLWETIKTTGKSIALNLLDKIKCKIAGGCPK | >128 | >128 | 128 | non-toxic |
| RaCa3T5KT8K | GLWEKIKKTGKSIALNLLDKIKCKIAGGCPP | >128 | >128 | 128 | non-toxic |
| RaCa3T8KS12K | GLWETIKKTGKKIALNLLDKIKCKIAGGCPP | ≥128 | >128 | >128 | non-toxic |
| RaCa3S12KG28K | GLWETIKTTGKKIALNLLDKIKCKIAGKCPP | >128 | >128 | >128 | non-toxic |
| RaCa3S12KP31K | GLWETIKTTGKKIALNLLDKIKCKIAGGCPK | ≥128 | >128 | >128 | non-toxic |
| RaCa3T8KT9K | GLWETIKKKGKSIALNLLDKIKCKIAGGCPP | >128 | >128 | >128 | non-toxic |
| RaCa3T8KA26R | GLWETIKKTGKSIALNLLDKIKCKIRGGCPP | >128 | >128 | >128 | non-toxic |
| RaCa3G28KT8K | GLWETIKKTGKSIALNLLDKIKCKIAGKCPP | >128 | >128 | >128 | non-toxic |
| RaCa3T8KT9KS12K | GLWETIKKKGKKIALNLLDKIKCKIAGGCPP | >128 | >128 | >128 | non-toxic |
| RaCa3G28KT8KS12K | GLWETIKKTGKKIALNLLDKIKCKIAGKCPP | ≥128 | >128 | ≥128 | toxic |
| RaCa3T5KT8KG28K | GLWEKIKKTGKSIALNLLDKIKCKIAGKCPP | >128 | >128 | >128 | non-toxic |
| RaCa3T8KT9KG28K | GLWETIKKKGKSIALNLLDKIKCKIAGKCPP | >128 | >128 | >128 | non-toxic |
| RaCa3G27KT8KT9K | GLWETIKKKGKSIALNLLDKIKCKIAKGCPP | >128 | >128 | >128 | non-toxic |
| RaCa3T8KT9KG27KG28K | GLWETIKKKGKSIALNLLDKIKCKIAKKCPP | >128 | >128 | >128 | non-toxic |
| RaCa3T5KT8KT9KS12KG27KG28K | GLWEKIKKKGKKIALNLLDKIKCKIAKKCPP | >128 | >128 | >128 | non-toxic |
| RaCa3S12KC23SC29S | GLWETIKTTGKKIALNLLDKIKSKIAGGSPP | >128 | >128 | >128 | non-toxic |
| RaCa3t1_29 | GLWETIKTTGKSIALNLLDKIKCKIAGGC | >128 | >128 | >128 | non-toxic |
| RaCa3S12Kt1_29 | GLWETIKTTGKKIALNLLDKIKCKIAGGC | 128 | >128 | >128 | non-toxic |
| RaCa3T8KT9KG27KG28Kt1_29 | GLWETIKKKGKSIALNLLDKIKCKIAKKC | ≥128 | >128 | >128 | non-toxic |
| RaCa3T5KT8KT9KS12KG27KG28Kt1_29 | GLWEKIKKKGKKIALNLLDKIKCKIAKKC | >128 | >128 | >128 | non-toxic |
| RaCa3T5KT8KS12Kt1_29 | GLWEKIKKTGKKIALNLLDKIKCKIAGGC | >128 | >128 | >128 | non-toxic |
| RaCa3C29St1_29 | GLWETIKTTGKSIALNLLDKIKCKIAGGS | >128 | >128 | >128 | non-toxic |
| RaCa3S12Kt1_23 | GLWETIKTTGKKIALNLLDKIKC | ≥128 | >128 | ≥128 | toxic |
| RaCa3T5KT8Kt1_23 | GLWEKIKKTGKSIALNLLDKIKC | >128 | >128 | >128 | non-toxic |
| RaCa3T5KT8KS12Kt1_23 | GLWEKIKKTGKKIALNLLDKIKC | 128 | >128 | >128 | non-toxic |
| RaCa3T5KT8KC23St1_23 | GLWEKIKKTGKSIALNLLDKIKS | >128 | >128 | >128 | non-toxic |
| RaCa3S12KC23SKt1_23 | GLWETIKTTGKKIALNLLDKIKS | >128 | >128 | >128 | non-toxic |
| RaCa3T5KT8KS12C23SKt1_23 | GLWEKIKKTGKKIALNLLDKIKS | >128 | >128 | >128 | non-toxic |
| RaCa7 | FFPRVLPLANKFLPTIYCALPKSVGN | >128 | >128 | >128 | non-toxic |
| RaCa7P7K | FFPRVLKLANKFLPTIYCALPKSVGN | 64 | 32-64 | 64 | toxic |
| RaCa7P7R | FFPRVLRLANKFLPTIYCALPKSVGN | 64 | 64 | 64 | toxic |
| RaCa7N10K | FFPRVLPLAKKFLPTIYCALPKSVGN | >128 | >128 | >128 | non-toxic |
| RaCa7T15K | FFPRVLPLANKFLPKIYCALPKSVGN | >128 | >128 | >128 | non-toxic |
| RaCa7Y17K | FFPRVLPLANKFLPTIKCALPKSVGN | >128 | >128 | >128 | non-toxic |
| RaCa7S23R | FFPRVLPLANKFLPTIYCALPKRVGN | >128 | >128 | >128 | non-toxic |
| RaCa7G25R | FFPRVLPLANKFLPTIYCALPKSVRN | >128 | >128 | >128 | non-toxic |
| RaCa7Y17KT15K | FFPRVLPLANKFLPKIKCALPKSVGN | >128 | >128 | >128 | non-toxic |
| RaCa7Y17KP7K | FFPRVLKLANKFLPTIKCALPKSVGN | >128 | >128 | ≥128 | non-toxic |
| RaCa7Y17KP14K | FFPRVLPLANKFLKTIKCALPKSVGN | >128 | >128 | >128 | non-toxic |
| RaCa7P7RY17K | FFPRVLRLANKFLPTIKCALPKSVGN | >128 | >128 | >128 | non-toxic |
| RaCa7P7KG25R | FFPRVLKLANKFLPTIYCALPKSVRN | 32-64 | 32 | 32 | toxic |
| RaCa7L8KG25R | FFPRVLPKANKFLPTIYCALPKSVRN | >128 | >128 | >128 | non-toxic |
| RaCa7P7KN10K | FFPRVLKLAKKFLPTIYCALPKSVGN | 64-128 | 64 | 64 | toxic |
| RaCa7P7RT15K | FFPRVLRLANKFLPKIYCALPKSVGN | 64 | 32 | 64 | toxic |
| RaCa7Y17KP7KT15K | FFPRVLKLANKFLPKIKCALPKSVGN | >128 | >128 | >128 | non-toxic |
| RaCa7Y17KP14KT15K | FFPRVLPLANKFLKKIKCALPKSVGN | >128 | >128 | >128 | non-toxic |
| RaCa7P7RY17KT15K | FFPRVLRLANKFLPKIKCALPKSVGN | >128 | 128 | >128 | non-toxic |
| RaCa7P7KG25RR4K | FFPKVLKLANKFLPTIYCALPKSVRN | 64 | 32 | 128 | toxic |
| RaCa7L8KG25RR4K | FFPKVLPKANKFLPTIYCALPKSVRN | >128 | >128 | >128 | non-toxic |
| RaCa7P7KN10KY17K | FFPRVLKLAKKFLPTIKCALPKSVGN | 128 | 128 | ≥128 | toxic |
| RaCa7t1_18 | FFPRVLPLANKFLPTIYC | >128 | >128 | >128 | non-toxic |
| RaCa7Y17KT15Kt1_18 | FFPRVLPLANKFLPKIKC | >128 | >128 | >128 | non-toxic |
| RaCa7Y17KP7Kt1_18 | FFPRVLKLANKFLPTIKC | >128 | >128 | >128 | non-toxic |
| RaCa7Y17KP14Kt1_18 | FFPRVLPLANKFLKTIKC | >128 | >128 | >128 | non-toxic |
| RaCa7P7RY17Kt1_18 | FFPRVLRLANKFLPTIKC | 128 | 128 | >128 | toxic |
| RaCa7P7KG25Rt1_18 | FFPRVLKLANKFLPTIYC | >128 | >128 | >128 | non-toxic |
| RaCa7L8KG25Rt1_18 | FFPRVLPKANKFLPTIYC | >128 | >128 | >128 | non-toxic |
| RaCa7P7KN10Kt1_18 | FFPRVLKLAKKFLPTIYC | 16 | 32 | 32-64 | toxic |
| RaCa7P7RT15Kt1_18 | FFPRVLRLANKFLPKIYC | 32 | 32 | 64 | toxic |
| RaCa7Y17KP7KT15Kt1_18 | FFPRVLKLANKFLPKIKC | 128 | >128 | >128 | non-toxic |
| RaCa7Y17KP14KT15Kt1_18 | FFPRVLPLANKFLKKIKC | >128 | >128 | >128 | non-toxic |
| RaCa7P7RY17KT15Kt1_18 | FFPRVLRLANKFLPKIKC | 128 | 128 | >128 | toxic |
| RaCa7P7KG25RR4Kt1_18 | FFPKVLKLANKFLPTIYC | >128 | >128 | >128 | non-toxic |
| RaCa7L8KG25RR4Kt1_18 | FFPKVLPKANKFLPTIYC | >128 | >128 | >128 | non-toxic |
| RaCa7P7KN10KY17Kt1_18 | FFPRVLKLAKKFLPTIKC | 64 | 64 | 64 | toxic |
| RaCa7P7RT15KY17KP14Kt1_18 | FFPRVLRLANKFLKKIKC | 32 | 16 | 16 | toxic |
| RaCa7P7RT15K1Y17KN10Rt1_18 | FFPRVLRLARKFLPKIKC | 64 | 16 | 64 | toxic |
| RaCa7P7RT15KY17KP3Rt1_18 | FFRRVLRLANKFLPKIKC | 16-32 | 16 | 16 | toxic |
| RaCa7P7RT15KC18St1_18 | FFPRVLRLANKFLPKIYS | >128 | >128 | >128 | non-toxic |
| RaCa7Y17KP14KC18St1_18 | FFPRVLPLANKFLKTIKS | >128 | >128 | >128 | non-toxic |
| RaCa7Y17KP14KT15KC18St1_18 | FFPRVLPLANKFLKKIKS | >128 | >128 | >128 | non-toxic |
| RaCa7P7RY17KT15KC18St1_18 | FFPRVLRLANKFLPKIKS | >128 | >128 | >128 | non-toxic |
| Ranatuerin4 | FLPFIARLAAKVFPSIICSVTKKC | 16 | 16 | 16 | toxic |
| RaCa1 | GLLDIIKTTGKDFAVKILDNLKCKLAGGCPP | >128 | >128 | >128 | non-toxic |
| RaCa1I5K | GLLDKIKTTGKDFAVKILDNLKCKLAGGCPP | >128 | >128 | >128 | non-toxic |
| RaCa1T8K | GLLDIIKKTGKDFAVKILDNLKCKLAGGCPP | >128 | >128 | >128 | non-toxic |
| RaCa1D12K | GLLDIIKTTGKKFAVKILDNLKCKLAGGCPP | >128 | >128 | >128 | non-toxic |
| RaCa1P31K | GLLDIIKTTGKDFAVKILDNLKCKLAGGCPK | >128 | >128 | >128 | non-toxic |
| RaCa1P31R | GLLDIIKTTGKDFAVKILDNLKCKLAGGCPR | >128 | >128 | >128 | non-toxic |
| RaCa1T8KD12K | GLLDIIKKTGKKFAVKILDNLKCKLAGGCPP | >128 | >128 | >128 | non-toxic |
| RaCa1D12KP31K | GLLDIIKTTGKKFAVKILDNLKCKLAGGCPK | >128 | >128 | >128 | non-toxic |
| RaCa1T8KG27K | GLLDIIKKTGKDFAVKILDNLKCKLAKGCPP | >128 | >128 | >128 | non-toxic |
| RaCa1T8KI5K | GLLDKIKKTGKDFAVKILDNLKCKLAGGCPP | >128 | >128 | >128 | non-toxic |
| RaCa1T8KN20K | GLLDIIKKTGKDFAVKILDKLKCKLAGGCPP | >128 | >128 | >128 | non-toxic |
| RaCa1P30RP31R | GLLDIIKTTGKDFAVKILDNLKCKLAGGCRR | >128 | >128 | >128 | non-toxic |
| RaCa1T8KI5KD12K | GLLDKIKKTGKKFAVKILDNLKCKLAGGCPP | >128 | >128 | >128 | non-toxic |
| RaCa1T8KI5KG27K | GLLDKIKKTGKDFAVKILDNLKCKLAKGCPP | >128 | >128 | >128 | non-toxic |
| RaCa1T8KN20KD19K | GLLDIIKKTGKDFAVKILKKLKCKLAGGCPP | ≥128 | >128 | >128 | non-toxic |
| RaCa1T8KN20KG27K | GLLDIIKKTGKDFAVKILDKLKCKLAKGCPP | >128 | >128 | >128 | non-toxic |
| RaCa1T8KI5KD12KP30RP31R | GLLDKIKKTGKKFAVKILDNLKCKLAGGCRR | >128 | >128 | >128 | non-toxic |
| RaCa1T8KN20KD19KP30RP31R | GLLDIIKKTGKDFAVKILKKLKCKLAGGCRR | ≥128 | >128 | >128 | non-toxic |
| RaCa1t1_29 | GLLDIIKTTGKDFAVKILDNLKCKLAGGC | >128 | >128 | >128 | non-toxic |
| RaCa1T8KN20KD19Kt1_29 | GLLDIIKKTGKDFAVKILKKLKCKLAGGC | 128 | 128 | >128 | toxic |
| RaCa1C29St1_29 | GLLDIIKTTGKDFAVKILDNLKCKLAGGS | >128 | >128 | >128 | non-toxic |
| RaCa1D12Kt1_23 | GLLDIIKTTGKKFAVKILDNLKC | >128 | >128 | >128 | non-toxic |
| RaCa1D12KD19Kt1_23 | GLLDIIKTTGKKFAVKILKNLKC | ≥128 | >128 | >128 | non-toxic |
| RaCa1T8KN20KD19Kt1_23 | GLLDIIKKTGKDFAVKILKKLKC | >128 | >128 | >128 | non-toxic |
| RaCa1C23St1_23 | GLLDIIKTTGKDFAVKILDNLKS | >128 | >128 | >128 | non-toxic |
| RaCa1T8KN20KD19KC23St1_23 | GLLDIIKKTGKDFAVKILKKLKS | >128 | >128 | >128 | non-toxic |
| LeBo1 | GIFSLIKGAAK | >128 | >128 | >128 | non-toxic |
| OdMa8 | GLISGILGAGKK | >128 | >128 | >128 | non-toxic |
| PeNi6 | AGLQFPVGRIHRHLKTR | >128 | >128 | >128 | non-toxic |
| PeNi12 | GAPKGCWTKSYPPKPCSGKR | >128 | >128 | >128 | non-toxic |
| PeNi13 | KEERGAPKGCWTKSYPPKPCSGKR | >128 | >128 | >128 | non-toxic |
| PeNi15 | FLPSSPWNEGTYVLKKLKS | >128 | >128 | >128 | non-toxic |
| PeNi17 | RMIRRPPGFSPFRVAPASSLKR | >128 | >128 | >128 | non-toxic |
| PeNi18 | RPRWSHRSRR | >128 | >128 | >128 | non-toxic |
| RaOm1 | GLLSGILGAGKK | >128 | >128 | >128 | non-toxic |
| RaSi2 | FPFPFGRR | >128 | >128 | >128 | non-toxic |
| AnFl1 | DNKWQNVHFHRSAVTGPTSFSFSHK | >128 | >128 | >128 | non-toxic |
| ApMe1 | VKCRVRR | >128 | >128 | >128 | non-toxic |
| ApMe2 | GAHKEVFKRDTALTKEAAKKAKK | >128 | >128 | >128 | non-toxic |
| ApMe3 | GWGLINIKIPPVLHKVSVPLVSKR | >128 | >128 | >128 | non-toxic |
| ApMe4 | KHHHIKLRHERHRRYILKSLI | >128 | >128 | >128 | non-toxic |
| ApMe5 | SILSTLSHKR | >128 | >128 | >128 | non-toxic |
| ApMe6 | RARKIRRRRGSLRHCVTIPSTPSGR | >128 | >128 | >128 | non-toxic |
| BoAr1 | AAGAGKVTKSAQKAQKAK | >128 | >128 | >128 | non-toxic |
| BoAr2 | ATAAECLKHPWLKIKK | >128 | >128 | >128 | non-toxic |
| BoAr3 | IIRATAAECLKHPWLKIKK | >128 | >128 | >128 | non-toxic |
| BoAr4 | SVASLAKNSAWPVSLKR | >128 | >128 | >128 | non-toxic |
| BoAr5 | VTISIARRVSSHKRG | >128 | >128 | >128 | non-toxic |
| BoCo1 | NKIKFINKYVKKVQLKKILVKS | >128 | >128 | >128 | non-toxic |
| CaCa3 | KHHHIKLRHGRHRRSVLRTLV | >128 | >128 | >128 | non-toxic |
| MiDe1 | VMLPKFKR | >128 | >128 | >128 | non-toxic |
| NaVi1 | TPLSDIFRGQLRSRVSR | >128 | >128 | >128 | non-toxic |
| NaVi2 | SSLSPLSSSSGLGKKKKRKSKRASR | >128 | >128 | >128 | non-toxic |
| NaVi4 | GSSSRSCRCIRLSRLSSKRT | >128 | >128 | >128 | non-toxic |
| PoSn1 | ISIKEALEHSFFHTVPRKWCKKH | >128 | >128 | >128 | non-toxic |
| PaVa1Q5K | RPRPKQVPPRPPHPRLRR | >128 | >128 | >128 | non-toxic |
| PaVa1Q6R | RPRPQRVPPRPPHPRLRR | >128 | >128 | >128 | non-toxic |
| PaVa1P2R | RRRPQQVPPRPPHPRLRR | >128 | >128 | >128 | non-toxic |
| PaVa1V7R | RPRPQQRPPRPPHPRLRR | >128 | >128 | >128 | non-toxic |
| PaVa1H13R | RPRPQQVPPRPPRPRLRR | >128 | >128 | >128 | non-toxic |
| PaVa1P4K | RPRKQQVPPRPPHPRLRR | >128 | >128 | >128 | non-toxic |
| PaVa1H13K | RPRPQQVPPRPPKPRLRR | >128 | >128 | >128 | non-toxic |
| PeNi4G4K | GLLKKVLGVGKKVLC | >128 | >128 | >128 | non-toxic |
| PeNi4V9R | GLLGKVLGRGKKVLC | >128 | >128 | >128 | non-toxic |
| PeNi4G10R | GLLGKVLGVRKKVLC | >128 | >128 | >128 | non-toxic |
| PeNi4V13K | GLLGKVLGVGKKKLC | >128 | >128 | >128 | non-toxic |
| CaCa2W22K | GLLGKVRGVGKKVLC | >128 | >128 | >128 | non-toxic |
| TeBi1P5K | KIKIKWGKVKDFLVGGMKAVGKK | >128 | >128 | >128 | non-toxic |
| TeBi1V14K | KIKIPWGKVKDFLKGGMKAVGKK | >128 | >128 | >128 | non-toxic |
| TeBi1G21K | KIKIPWGKVKDFLVGGMKAVKKK | >128 | >128 | >128 | non-toxic |
| TeBi1I4K | KIKKPWGKVKDFLVGGMKAVGKK | >128 | >128 | >128 | non-toxic |
| TeBi1A19K | KIKIPWGKVKDFLVGGMKKVGKK | >128 | >128 | >128 | non-toxic |
| TeBi1D11K | KIKIPWGKVKKFLVGGMKAVGKK | >128 | >128 | >128 | non-toxic |
| TeBi1W6K | KIKIPKGKVKDFLVGGMKAVGKK | >128 | >128 | >128 | non-toxic |
| TeBi1V9K | KIKIPWGKKKDFLVGGMKAVGKK | >128 | >128 | >128 | non-toxic |
| TeRu4S6K | SWLSKKVKKLVNKKNYTRLEKLAKKKLFNE | >128 | >128 | >128 | non-toxic |
| TeRu4F28K | SWLSKSVKKLVNKKNYTRLEKLAKKKLKNE | >128 | >128 | >128 | non-toxic |
| TeRu4N29K | SWLSKSVKKLVNKKNYTRLEKLAKKKLFKE | >128 | >128 | >128 | non-toxic |
| TeRu4S1R | RWLSKSVKKLVNKKNYTRLEKLAKKKLFNE | >128 | >128 | >128 | non-toxic |
| TeRu4N15K | SWLSKSVKKLVNKKKYTRLEKLAKKKLFNE | >128 | >128 | >128 | non-toxic |
| AmMa1T5K | GILDKLKQLGKAAVQGLLSKAACKLAKTC | >128 | >128 | >128 | non-toxic |
| AmMa1Q8K | GILDTLKKLGKAAVQGLLSKAACKLAKTC | >128 | >128 | >128 | non-toxic |
| AmMa1A12K | GILDTLKQLGKKAVQGLLSKAACKLAKTC | >128 | >128 | >128 | non-toxic |
| AmMa1A21K | GILDTLKQLGKAAVQGLLSKKACKLAKTC | >128 | >128 | >128 | non-toxic |
| ApCe1L22R | GIYTGRLLPVYIPQPRPPHPRRRR | >128 | >128 | >128 | non-toxic |
| ApCe1Y3R | GIRTGRLLPVYIPQPRPPHPRLRR | >128 | >128 | >128 | non-toxic |
| ApCe1G5R | GIYTRRLLPVYIPQPRPPHPRLRR | >128 | >128 | >128 | non-toxic |
| ApCe1V10R | GIYTGRLLPRYIPQPRPPHPRLRR | >128 | >128 | >128 | non-toxic |
| ApCe1P9R | GIYTGRLLRVYIPQPRPPHPRLRR | >128 | >128 | >128 | non-toxic |
| CaCa2D21K | FIKTQVLKHLVAGVRVARGLKWKWR | >128 | >128 | >128 | non-toxic |
| CaCa2T4K | FIKKQVLKHLVAGVRVARGLDWKWR | >128 | >128 | >128 | non-toxic |
| CaCa2W22K | FIKTQVLKHLVAGVRVARGLDKKWR | >128 | >128 | >128 | non-toxic |
| CaCa2H9K | FIKTQVLKKLVAGVRVARGLDWKWR | >128 | >128 | >128 | non-toxic |
| CaCa2A12K | FIKTQVLKHLVKGVRVARGLDWKWR | >128 | >128 | >128 | non-toxic |
| CaCa4A6R | RRFFFRTAPCGYSRKFCKITRRKR | >128 | >128 | >128 | non-toxic |
| CaCa4T7R | RRFFFARAPCGYSRKFCKITRRKR | >128 | >128 | >128 | non-toxic |
| CaCa4F5K | RRFFKATAPCGYSRKFCKITRRKR | >128 | >128 | >128 | non-toxic |
| CaCa4C10R | RRFFFATAPRGYSRKFCKITRRKR | >128 | >128 | >128 | non-toxic |
| MyGu1A6K | RRAIFKSIRGYLGLRKR | >128 | >128 | >128 | non-toxic |
| MyGu1Y11K | RRAIFASIRGKLGLRKR | >128 | >128 | >128 | non-toxic |
| MyGu1L14K | RRAIFASIRGYLGKRKR | >128 | >128 | >128 | non-toxic |
| MyGu1G13K | RRAIFASIRGYLKLRKR | >128 | >128 | >128 | non-toxic |
| MyGu1A3K | RRKIFASIRGYLGLRKR | >128 | >128 | >128 | non-toxic |
| NaVi3L4K | KLFKTLWKLKR | >128 | >128 | >128 | non-toxic |
| NaVi3T5R | KLFLRLWKLKR | >128 | >128 | >128 | non-toxic |
| NaVi3W7K | KLFLTLKKLKR | >128 | >128 | >128 | non-toxic |
| NaVi3L6R | KLFLTRWKLKR | >128 | >128 | >128 | non-toxic |
| OdMa2G19K | GLLRGILGAGKKIVCGLSKLC | >128 | >128 | >128 | non-toxic |
| OdMa2G16K | GLLRGILGAGKKIVCKLSGLC | >128 | >128 | >128 | non-toxic |
| OdMa2L3R | GLRRGILGAGKKIVCGLSGLC | >128 | >128 | >128 | non-toxic |
| OdMa2G5R | GLLRRILGAGKKIVCGLSGLC | 128 | 128 | 32-64 | toxic |
| OdMa2A9R | GLLRGILGRGKKIVCGLSGLC | >128 | >128 | >128 | non-toxic |
| OdMa2C15K | GLLRGILGAGKKIVKGLSGLC | >128 | >128 | >128 | non-toxic |
| OdMa2S18K | GLLRGILGAGKKIVCGLKGLC | >128 | >128 | >128 | non-toxic |
| OdMa12T5K | GFMDKAKNVAKNVAVTLLYNLKCKITKAC | >128 | >128 | >128 | non-toxic |
| OdMa12N20K | GFMDTAKNVAKNVAVTLLYKLKCKITKAC | >128 | >128 | >128 | non-toxic |
| LiVe2I5K | GWLDKAKKVASVVAGLGKR | >128 | >128 | >128 | non-toxic |
| LiVe2S11K | GWLDIAKKVAKVVAGLGKR | >128 | >128 | >128 | non-toxic |
| LiVe2A14K | GWLDIAKKVASVVKGLGKR | >128 | >128 | >128 | non-toxic |
| DeNo1001 | DLLSGLGKAAKKVAKTVLKNLLKC | >128 | >128 | >128 | non-toxic |
| DeNo1002 | NLLDTLKNLAKKLAKKLLKKLLKKL | 64-128 | 128 | 128 | toxic |
| DeNo1003 | NLLSTLLDAAKKAAKGAAKSAAKKLAKKLAKKL | >128 | >128 | >128 | non-toxic |
| DeNo1004 | HLLSGLLSAAKKAAKKAAKKALKKLLKKLLKKL | >128 | >128 | >128 | non-toxic |
| DeNo1005 | GLFSLLKKLLKKLLKKLLKKLLKKLLKKL | >128 | >128 | >128 | non-toxic |
| DeNo1006 | NLLDTLKKKAKKVAKKVLKKLLKKLLKKL | >128 | >128 | >128 | non-toxic |
| DeNo1007 | FLPSIIKGAAKKLPKIFCKILKKC | 128->128 | >128 | >128 | non-toxic |
| DeNo1008 | GLLSLLKKLLKKLLKKLLKKL | 8.0-16.0 | 32 | 16 | toxic |
| DeNo1009 | DLLKTLGKAAKKAAKTALKAALKGLLKKLAKKL | >128 | >128 | >128 | non-toxic |
| DeNo1010 | VLGGLLKKLLKKLLKKL | 64 | 128 | 128 | toxic |
| DeNo1011 | HLLSLLKKAAKKLLKKLLKKLAKKL | 32 | 32 | 32-64 | toxic |
| DeNo1012 | CLLDTLKCVAKGVAGTLLDTLKCKITGKC | >128 | >128 | >128 | non-toxic |
| DeNo1013 | KIFGKILKKLLKKLLKKLLKKL | 64-128 | 64 | 64 | toxic |
| DeNo1014 | ALPSLLKKLAKKLAKKLLKKLLKKLLKKLLKKL | >128 | 128->128 | 128->128 | toxic |
| DeNo1015 | NLLDTLKNVAKNVAKNVLDTLKCKITCKC | >128 | >128 | >128 | non-toxic |
| DeNo1016 | FLPIIAGLAAKFLPKIFCKITKKC | 4 | 8-16 | 8 | toxic |
| DeNo1017 | FLPIIAGLAAKLLPKLFCKITKKC | 8-16 | 16 | 16 | toxic |
| DeNo1018 | WLPKIAGKIAGKLLKKLLKKIKKK | 128 | 128 | 128 | toxic |
| DeNo1019 | FLPKIAGKAAKKLPKIFCKITKKC | >128 | >128 | >128 | non-toxic |
| DeNo1020 | TLPDVAKNVAKNVAKTVLDTLKCKITGKC | >128 | >128 | >128 | non-toxic |
| DeNo1021 | KLFGKLLKKLKKILKKIAKKIKKKL | >128 | >128 | >128 | non-toxic |
| DeNo1022 | GLLSLLKKIGKKIGKLL | 128 | >128 | >128 | non-toxic |
| DeNo1023 | DLLKTLKKIAKKLLKTLLKKLLKKLLKKL | >128 | >128 | >128 | non-toxic |
| DeNo1024 | KLFGKILGKIAKKILGKILGALLSKLLSAL | >128 | >128 | >128 | non-toxic |
| DeNo1025 | DLLSCLKKKGKCVLKNL | >128 | >128 | >128 | non-toxic |
| DeNo1026 | RLPSLFKKLFKKIAKVVGKIAKKILKK | 128 | 128->128 | 128->128 | toxic |
| DeNo1027 | RLPSIIPGIAGKLGGLLGGLLKGL | >128 | >128 | >128 | non-toxic |
| DeNo1028 | CLPSLLPSLFKKL | >128 | >128 | >128 | non-toxic |
| DeNo1029 | SLPSILSGIAGKL | >128 | >128 | >128 | non-toxic |
| DeNo1030 | RLPRIFRGIRGKL | >128 | >128 | >128 | non-toxic |
| DeNo1031 | PLPPIIPGIAGKLLGGLLGLLKKL | 128 | 128 | 128 | toxic |
| DeNo1032 | YLPSVLPSVLKPL | >128 | >128 | >128 | non-toxic |
| DeNo1033 | PLPPIIPGLASGLLSGLC | >128 | >128 | >128 | non-toxic |
| DeNo1034 | KLPSIIKAAAKALPKLF | >128 | >128 | >128 | non-toxic |
| DeNo1035 | QLPRIAGKIAKKL | >128 | >128 | >128 | non-toxic |
| DeNo1036 | QLPSVLPAIAKAL | >128 | >128 | >128 | non-toxic |
| DeNo1037 | CLPSILC | >128 | >128 | >128 | non-toxic |
| DeNo1038 | MLPSIAGAAAKGLPKLFCKITKKC | >128 | >128 | >128 | non-toxic |
| DeNo1039 | MLPKIFGKIFKKILKKILKKILKKILKKLLKKL | 32 | 64 | 32 | toxic |
| DeNo1040 | MLPSILGALLKLL | >128 | >128 | >128 | non-toxic |
| DeNo1041 | MLPKIAGKIAKKL | >128 | >128 | >128 | non-toxic |
| DeNo1042 | MLPKIAGAIAKLL | >128 | >128 | >128 | non-toxic |
| DeNo1043 | WLPKIAGKIAGKL | >128 | >128 | >128 | non-toxic |
| DeNo1044 | CLPSILCKITKKC | >128 | >128 | >128 | non-toxic |
| DeNo1045 | FLPKIFKKIAKKL | >128 | >128 | >128 | non-toxic |
| DeNo1046 | VLGSLLKGLLKKL | >128 | >128 | >128 | non-toxic |
| DeNo1047 | ALPSIIKGLLKKL | >128 | >128 | >128 | non-toxic |
| DeNo1048 | LLPSLLKGLLKKL | >128 | >128 | >128 | non-toxic |
| DeNo1049 | ALLSLLKKLLKKL | >128 | >128 | >128 | non-toxic |
| DeNo1050 | FLPKIAGKIAGKL | >128 | >128 | >128 | non-toxic |
| DeNo1051 | ALPSLLKKLLKKL | >128 | >128 | >128 | non-toxic |
| DeNo1052 | YLPSVLKGLLKKL | >128 | >128 | >128 | non-toxic |
| DeNo1053 | LLPSLLKGLAKKL | >128 | >128 | >128 | non-toxic |
| DeNo1054 | QLPKIAGKIAKKL | >128 | >128 | >128 | non-toxic |
| DeNo1055 | FLPKIFKKIAKKI | >128 | >128 | >128 | non-toxic |
| DeNo1056 | GLLSLLKKLLKKL | >128 | >128 | >128 | non-toxic |
| DeNo1057 | ILGKLLKKLLKKL | >128 | >128 | >128 | non-toxic |
| DeNo1058 | FLPKIAGKIAKKL | >128 | >128 | >128 | non-toxic |
| RaCa2 | FFPIIARLAAKVIPSLVCAVTKKC | 8 | 32 | 16 | toxic |
| RaCa2I4K | FFPKIARLAAKVIPSLVCAVTKKC | 128 | 128 | 128 | toxic |
| RaCa2P14K | FFPIIARLAAKVIKSLVCAVTKKC | 8 | 4-8 | 16 | toxic |
| RaCa2S15K | FFPIIARLAAKVIPKLVCAVTKKC | 16 | 16 | 16 | toxic |
| RaCa2A19K | FFPIIARLAAKVIPSLVCKVTKKC | 32 | 32 | 32-64 | toxic |
| RaCa2A19KI4K | FFPKIARLAAKVIPSLVCKVTKKC | 128 | 128 | 128 | toxic |
| RaCa2S15KA19KT21K | FFPIIARLAAKVIPKLVCKVKKKC | 128 | 128 | 128 | toxic |
| RaCa2I4KT21KA19K | FFPKIARLAAKVIPSLVCKVKKKC | 128 | 128 | 128 | toxic |
| RaCa2A19KI4KP3R | FFRKIARLAAKVIPSLVCKVTKKC | 32 | 32 | 64 | toxic |
| RaCa2C24S | FFPIIARLAAKVIPSLVCAVTKKS | 32 | 16 | 32 | toxic |
| RaCa2C18SC24S | FFPIIARLAAKVIPSLVSAVTKKS | 128 | 128 | 128 | toxic |
| RaCa2t1_18 | FFPIIARLAAKVIPSLVC | 64 | 64 | 32-64 | toxic |
| RaCa2P3RS15Kt1_18 | FFRIIARLAAKVIPKLVC | 16 | 8 | 16-32 | toxic |
| RaCa2I4KS15Kt1_18 | FFPKIARLAAKVIPKLVC | 32 | 16 | 64 | toxic |
| RaCa2P3RI4KS15Kt1_18 | FFRKIARLAAKVIPKLVC | 16 | 8 | 16 | toxic |
| RaCa2C18St1_18 | FFPIIARLAAKVIPSLVS | ≥128 | >128 | >128 | non-toxic |
| RaCa2P3RI4KS15C18SKt1_18 | FFRKIARLAAKVIPKLVS | 128 | 128 | >128 | toxic |

**References**

Chung, J., Gulcehre, C., Cho, K., & Bengio, Y. (2014, December 11). *Empirical Evaluation of Gated Recurrent Neural Networks on Sequence Modeling*. arXiv. Retrieved from http://arxiv.org/abs/1412.3555
